# Estimation of parameters for the modified Weibull distribution under step-stress accelerated life testing with application to aircraft windshields data

**DOI:** 10.1038/s41598-025-95469-x

**Published:** 2025-07-11

**Authors:** Mohammed M. A. Almazah, Mohammed A. Alshahrani, Qasim Ramzan, Irfan Ali Raza, Muhammad Tahir, Zahoor Ahmad

**Affiliations:** 1 Department of Mathematics, College of Sciences and Arts (Muhyil), King Khalid University, 61421 Muhyil, Saudi Arabia; 2 Department of Mathematics, College of Science and Humanities in Al-Kharj, Prince Sattam bin Abdulaziz University, 11942 Al-Kharj, Saudi Arabia; 3 Department of Statistics, Government Graduate College Jauharabad, Khushab, Punjab Pakistan; 4 Department of Statistics, University of Sargodha, Sargodha, Pakistan; 5 Department of Statistics, Government College University Faisalabad, Faisalabad, Pakistan; 6 College of Statistical Sciences, University of Punjab, Lahore, Pakistan

**Keywords:** Accelerated life testing, Balanced loss function, Fisher information, Modified Weibull Distribution, Progressive Type-II Censoring, Statistics, Applied mathematics

## Abstract

In the field of engineering, precise estimation and reliability analysis are vital for ensuring the safety and performance of systems. This research employs several advanced statistical techniques to achieve these objectives. The Modified Weibull Distribution (MWD), a variant of the type-II Weibull model introduced by Lai et al. (IEEE Trans Reliab 52 (1):33–37, 2003), has gained significant attention in engineering and technology for its versatility in modeling bathtub-shaped failure rate data. Its simple and flexible failure rate function (FRF) facilitates straightforward parameter estimation using various methods. This paper introduces novel techniques for parameter estimation in step-stress partially accelerated life testing (SSPALT) for the MWD, a key aspect for predicting component lifespan under varying stress conditions. We compare Maximum Likelihood Estimators (MLE) and Bayesian estimators, utilizing gamma priors due to their suitability for capturing the positive, skewed nature of failure rate parameters, and evaluate these across balanced and unbalanced loss functions, such as Square Error, General Entropy, and Linear Exponential. Through simulation studies, we assess the performance of these estimators and validate our approach using failure times of aircraft windshield data. This work offers practical tools for reliability engineers to improve maintenance scheduling, risk assessment, and the overall reliability of engineering systems.

## Introduction

Understanding the formation and interpretation of probability distributions is fundamental to modeling naturally occurring random phenomena. These distributions provide critical insights into event durations and occurrence probabilities, aiding in the comprehension of real-world processes. Commonly used distributions, such as the exponential, Weibull, Modified Weibull Distribution (MWD), and normal distributions, are frequently employed to estimate these characteristics. However, real-world data often deviates from these standard patterns, necessitating the exploration of diverse and specialized distributions to enhance our understanding of phenomena like the reliable operational lifespan of machinery or the frequency of specific events. This pursuit of novel probability models improves our ability to predict and interpret complex systems with greater accuracy.

Accelerated Life Testing (ALT) is a pivotal methodology in reliability engineering, where test units are deliberately subjected to harsher conditions such as elevated force, temperature, humidity, or voltage than those encountered during normal operation. By simulating years of wear in a condensed timeframe, ALT enables the rapid and cost-effective collection of failure time data, significantly reducing testing duration and expenses compared to traditional methods. This approach is particularly valuable in industries such as electronics, automotive, and aerospace, where high reliability is paramount. Through ALT, engineers gain essential insights into component performance under extreme conditions, facilitating the design of more robust and durable products for everyday use.

The data obtained from ALT is typically analyzed using a life-stress model to predict product performance under standard operating conditions. This model establishes a relationship between stress levels and expected lifespan. However, in some cases, defining an precise life-stress model is challenging, rendering traditional ALT less effective for predicting performance in typical conditions. In such scenarios, Partially Accelerated Life Testing (PALT) emerges as a practical alternative for reliability analysis of highly reliable units, components, or systems. According to^2^, PALT can apply stress in various forms, with Step-Stress PALT (SSPALT) and Constant-Stress PALT (CSPALT) being two prevalent techniques. In SSPALT, test units begin under normal conditions; if no failure occurs within a specified period, stress is incrementally increased until failure or censoring occurs, providing data on responses to escalating stress. Conversely, CSPALT subjects each unit to a fixed stress level either normal or accelerated throughout the test duration, simplifying the procedure and enabling straightforward reliability comparisons across units. Both methods offer distinct advantages, with their application depending on the specific requirements of the reliability analysis. Numerous studies have explored PALT under conventional censoring schemes, including works by^3–14^. These investigations typically employ various censoring techniques to evaluate reliability data. Additionally, Ismail^15^ has focused on SSPALT with Progressive Type-II censoring (PT-II), allowing dynamic stress adjustments based on unit performance. CSPALT has also been studied with traditional censoring by^16^ and^17^, and with progressive censoring schemes by^18^.

While estimates from censored data are generally less precise than those from complete data, they offer significant cost and time savings, making censoring schemes like Type-I (time-restricted) and Type-II (failure-order) widely adopted. Progressive censoring, such as PT-II with Constant-Barrier Removals (PT-II CBRs), is particularly effective when intermediate removals occur due to external factors, offering flexibility in real-world testing scenarios. This approach has been examined by^19–23^.

Despite these advancements, a notable gap exists in the literature: no studies have addressed parameter estimation for the MWD under SSPALT with PT-II CBRs. This paper aims to fill this gap by evaluating the applicability of Maximum Likelihood Estimation (MLE) versus Bayesian estimation for MWD parameters in this context. We employ both symmetric Squared Error Loss Function (SELF) and asymmetric loss functions, including the Linear-Exponential (LINEX) loss function^24^ and the General Entropy Loss Function (GELF). Building on^17^’s foundational work, we model the heterogeneous nature of reliability processes using a three-component mixture of power distributions. Bayesian inference is developed, producing estimators under various loss functions, which are analyzed across different test termination times and sample sizes. Monte Carlo simulations and real-world data applications validate our findings through posterior risk analysis, assessing estimator performance. These contributions are significant as they introduce a novel framework for reliability analysis, enhancing predictive accuracy for high-reliability systems under complex censoring schemes an area with broad implications for engineering and statistical science.

The following is an outline of this article’s structure: We give a thorough explanation of the MW distribution and the $\hbox {S}SSPALT$ model under $PT\text {-}II CBRs$ in section “Model perturbation”. The *MLE* and confidence interval (*CI*) estimators under *SSPALT* are introduced in section “Likelihood estimation of model parameters”. Furthermore, to determine the maximum a posterior (*MAP*) and full Bayesian point and credible intervals estimation of the model parameters, we use the metropolis-hasting (*MH*) sampling technique in section “Bayesian estimation”. Section “Numerical evaluation” presents the simulation findings and an application to real data, which shed light on the functionality and behavior of the considered model. The paper is finally brought to a close in section “Concluding remarks and future directions”, which concludes the results and suggests possible directions for future study.

## Model perturbation

### Modified Weibull distribution (*MWD*): a lifetime model

The lifespan of a test object, denoted as $X_{1}$, is presumed to follow *MWD*, which is a type-II Weibull model discussed by^1^, which garners interest from practitioners and researchers due to its straightforwardness, suitability to model the data with bathtub-shaped failure rate, and adaptability of FRF. The probability density function (*PDF*) of *MWD* is defined as:

1 $$\begin{aligned} f (t)= \beta t^{\zeta -1} (\zeta +\phi t)\exp \left\{ -\beta t^{\zeta }e^{\phi t}+\phi t \right\} , \end{aligned}$$

where $t\ge 0.$ And $\beta \ge 0$, $\phi \ge 0$ are the scale parameter, controlling the spread of the distribution (larger $\beta$ compresses the time scale). $\zeta \ge 0$ is the shape parameter, affecting the distribution’s form. The survival function (*SF*) is expressed as $S (t) = 1- F (t)$, where *F* (*t*) is the *CDF*. The failure rate, denoted as *h* (*t*), is defined as the rate at which a component experiences failures, presented in failures per time unit, and can be explicitly given by the formula $h (t) = f (t) / S (t)$. Hence, for our model we have:

2 $$\begin{aligned} S (t)= \exp \left\{ -\beta t^{\zeta }e^{\phi t } \right\} \end{aligned}$$

3 $$\begin{aligned} h (t)= \beta t^{\zeta -1} (\zeta +\phi t)e^{\phi t} \end{aligned}$$

As the *CDF* for the *MWD* is not in closed form. So, generation of samples was performed using the root-finding approach. We employed a well-established numerical root-finding technique, specifically the uniroot function, to determine quantiles. This method ensures accurate sampling by solving for the value of *t* that satisfies the equation $F (t) = p$, where *p* is a uniform random variable drawn from *U* (0, 1) . This approach is widely accepted in statistical literature for generating samples from complex distributions and is a standard alternative to direct inversion methods. The technique is robust and provides highly accurate results when applied with appropriate parameter ranges.

#### Stepped-stress plan accelerated life testing (*SSPALT*)

The testing procedure of *SSPALT* and its underlying assumptions are outlined below.

*Testing procedure*

i A sample of *n* identical test units is subjected to the life test, with the lifespan of each test unit following the *MWD*.
ii Initially, *n* units undergo normal use conditions until a predetermined time $\varrho$. If no failures occur, they are then subjected to accelerated conditions (stress).

Let *W* and *X* represent the random variables denoting the lifespan of a test item under normal conditions and accelerated conditions, respectively. Within the context of the tampered random variable (*TRV*) model as given by^14^, the lifespan of a test unit undergoing *SSPALT* can be expressed as:

4 $$\begin{aligned} X= \left\{ \begin{array}{ll} W,& \quad \text {if } W\le \varrho , \\ \varrho + \rho ^{-1} (W-\varrho ),& \quad \text {if } W>\varrho . \end{array} \right. \end{aligned}$$

where $\varrho \ge 0$ is the time of stress change, and $\rho >1$ is known as acceleration factor. Hence, the *PDF* of the *MWD* under *SSPALT* becomes

5 $$\begin{aligned} f (x)= \left\{ \begin{array}{ll} f_{1} (x)=\beta x^{\zeta -1} (\zeta +\phi x)\exp \left\{ -\beta x^{\zeta }e^{\phi x}+\phi x \right\} & \quad 0<x\le \varrho , \\ f_{2} (x)=\beta \rho \wp _{\rho x }^{\zeta -1}\left ( \zeta +\phi \wp _{\rho x } \right) \exp \left\{ -\beta \wp _{\rho x }^{\zeta }e^{ \phi \wp _{\rho x }}+\phi \wp _{\rho x } \right\} & \quad x>\varrho , \end{array} \right. \end{aligned}$$

where $\wp _{\rho x }=\varrho +\rho (x-\varrho )$. The corresponding *SF* can take the form

6 $$\begin{aligned} S (x)= \left\{ \begin{array}{ll} S_{1} (x)=\exp \left\{ -\beta x^{\zeta }e^{\phi x} \right\} & \quad 0<x\le \varrho , \\ S_{2} (x)=\exp \left\{ -\beta \wp _{\rho x }^{\zeta }e^{\phi \wp _{\rho x }} \right\} & \quad x>\varrho , \end{array} \right. \end{aligned}$$

Let’s consider a scenario where we have *n* test items undergoing test under normal conditions to determine the breaking stress of carbon fibers, which follows the *MWD*. Upon observing the first failure at $X_{1}, R_{1}$ units, if no failure occurs or if units are removed from experiment before a specified time $\varrho$, they are transitioned to accelerated conditions. The removal probability of a unit, denoted as p , remains constant across all breaking stress data. Similarly, upon observing the second failure at $X_{2}, R_{2}$ removals of units are assumed to occur. This procedure continues til the observation of $m_{th}$ breaking stress unit, at which point the experiment concludes. This method results in obtaining a $PT\text {-}II CBRs$ sample represented as $(X_{1}, R_{1}), (X_{2}, R_{2}), (X_{3}, R_{3}), \ldots , (X_{m}, R_{m})$. It’s important to note that $X_{1}< X_{2}< X_{3}, \ldots < X_{m}$. The number of items removed at the $i^{th}$ stage, denoted as $R_{i}$, follows a binomial distribution (see e.g.^23^). Considering $n_{1}$ as the number of failure units observed before time $\varrho$ under normal conditions, and $(m - n_{1})$ as the number of failed units observed after $\varrho$ under stress conditions, the observations in *SSPALT* under $PT\text {-}II CBR$ are structured as $(X_{1}, R_{1})< (X_{2}, R_{2})< (X_{3}, R_{3}),< (X_{{n}_{1}}, R_{{n}_{1}})< \varrho< (X_{{n}_{1}}+1, R_{{n}_{1}}+1),< \ldots , (X_{m}, R_{m})$, assuming the number of items removed, denoted as $R_{1} = r_{1}, R_{2} = r_{2}, R_{3} = r_{3}, \ldots , R_{m} = r_{m}$, remains fixed.

##  Likelihood estimation of model parameters

### Maximum likelihood estimation

The following section delves into the process of estimating *MLE* for unknown parameters using $PT\text {-}II CBR$ data within the *SSPALT* model. Furthermore, it investigates the confidence limits of these parameters through asymptotic *CI* estimation methods. In the context of this discussion, let $\Phi = (\zeta ,\phi ,\beta ,\rho )$ represent the set of parameters. Subsequently, the conditional likelihood can be written as detailed in previous works such as^25–30^. Hence, we have

7 $$\begin{aligned} L (\Phi ;x|R=r)&= f_{X_{1},\ldots ,X_{n_{1}},X_{n_{1}+1}\ldots X_{m}} (x_{1},\ldots ,x_{n_{1}},x_{n_{1}+1}\ldots x_{m}) \nonumber \\&= k\prod _{i=1}^{n_{1}}f_{1} (x_{i}) \left[ S_{1} (x_{i}) \right] ^{r_{i}}\prod _{i=n_{1}+1}^{m}f_{2} (x_{i}) \left[ S_{2} (x_{i}) \right] ^{r_{i}} \end{aligned}$$

where, *k* is a constant that is independent of the parameters, $n = m + \sum _{i=1}^{m}r_{i}$, $1 \le i\le m,$ $0<x_{1}<\cdots<x_{n_{1}},x_{n_{1}+1}<\cdots<x_{m}<\infty , n, m \in N,r_{i}\sim B (n-m- \sum _{l=0}^{i-1}r_{i},p)$ for $i = 1,2,3,\ldots m - 1$, $r_{0} = 0$, $c = \prod _{i=1}^{m}\xi _{i}$ with $\xi _{i} = \sum _{j=i}^{m} (r_{j}+1)$ and for $\xi _{1}= n$. Substituting $f_{1} (x_{i}), f_{2} (x_{i}), S_{1} (x_{i})$ and $S_{2} (x_{i})$ from Eqs.  (5) and (6) into (7), it reduces to

8 $$\begin{aligned} L (\Phi ;x_{i}|R)&= k\beta ^{m}\rho ^{m-n_{1}}\prod _{i=1}^{n_{1}} \left[ x_{i}^{\zeta -1} (\zeta +\phi x_{i}) \exp \left\{ -\beta x_{i}^{\zeta }e^{\phi x_{i}}+\phi x_{i} \right\} \exp \left\{ -\beta r_{i} x_{i}^{\zeta }e^{\phi x_{i}} \right\} \right] \nonumber \\ &\quad \times \left[ \prod _{i=n_{1}+1}^{m} \wp _{{{{\rho x_{i}}}}} ^{\zeta -1} \left ( \zeta +\phi \wp _{{{{\rho x_{i}}}}} \right) \exp \left\{ -\beta \wp _{{{{\rho x_{i}}}}}^{\zeta }e^{\phi \wp _{{{{\rho x_{i}}}}}}+\phi \wp _{{{{\rho x_{i}}}}} \right\} \exp \left\{ -\beta r_{i} \wp _{{{{\rho x_{i}}}}}^{\zeta }e^{\phi \wp _{{{{\rho x_{i}}}}}} \right\} \right] \end{aligned}$$

As previously mentioned, the number of removed items $R_{i},$ from experiment are independent binomial random variables with a probability of *p* at every stage.Hence,

9 $$\begin{aligned} P ( R_{1}=r_{1};p)= \left ( {\begin{array}{c}n-m\\ r_{1}\end{array}}\right) p^{r_{1}} (1-p)^{ (n-m-r_{1})} \end{aligned}$$

for $$i = 2, 3,\ldots , m-1,$$

10 $$\begin{aligned} P ( R_{i};p)&= P ( R_{i}=r_{i}| R_{i-1}=r_{i-1},\ldots , R_{1}=r_{1})\nonumber \\&= \left ( {\begin{array}{c}n-m-\sum _{ s=0}^{i-1}r_{s} \\ r_{i}\end{array}}\right) p^{ri} (1-p)^{ (n-m-\sum _{ s=0}^{i-1}r_{s})} \end{aligned}$$

we assume that removals, $R_{i,s},$ and $X_{i}$ are independent. Thus, full likelihood is

11 $$\begin{aligned} L (\Phi , p; x)= L (\Phi ; x|R = r) P (R = r; p) \end{aligned}$$

and

12 $$\begin{aligned} P (R = r; p)&= P ( R_{1}=r_{1}) P ( R_{2}=r_{2}| R_{1}=r_{1}) P ( R_{3}=r_{3}|R_{2}=r_{2},R_{1}=r_{1}),\nonumber \\ &\quad \dots ,P ( R_{m-1}=r_{m-1}| R_{m-2}=r_{m-2},\ldots , R_{1}=r_{1}) \end{aligned}$$

Using Eqs. (9) where (10) in (12), produce

13 $$\begin{aligned} P (R = r; p)&= \dfrac{ (n-m)!p^{\sum _{ i=0}^{m-1}r_{i}} (1-p)^{ (m-1) (n-m)-\sum _{ i=0}^{m-1} (m-i)r_{i}}}{ \left ( n-m-\sum _{ i=0}^{m-1}r_{i}\right) !\prod _{s=1}^{i-1}r_{s}!} \end{aligned}$$

Now, using Eqs. (8), (11) and (13), the full likelihood function can be written as

14 $$\begin{aligned} L (x|\Phi )&= k\beta ^{m}\rho ^{m-n_{1}}\prod _{i=1}^{n_{1}} x_{i}^{\zeta -1} (\zeta +\phi x_{i}) \exp \left\{ -\beta \sum _{i=1}^{n_{1}} x_{i}^{\zeta }e^{\phi x_{i}}+\phi \sum _{i=1}^{n_{1}} x_{i} \right\} \exp \left\{ -\beta \sum _{i=1}^{n_{1}}r_{i} x_{i}^{\zeta }e^{\phi x_{i}} \right\} \nonumber \\ &\quad \times \prod _{i=n_{1}+1}^{m} \wp _{{{{\rho x_{i}}}}} ^{\zeta -1}\left ( \zeta +\phi \wp _{{{{\rho x_{i}}}}}\right) \exp \left\{ -\beta \sum _{i=n_{1}+1}^{m} \wp _{{{{\rho x_{i}}}}}^{\zeta }e^{\phi \wp _{{{{\rho x_{i}}}}}}+\phi \sum _{i=n_{1}+1}^{m}\wp _{{{{\rho x_{i}}}}} \right\} \nonumber \\ &\quad \times \exp \left\{ -\beta \sum _{i=n_{1}+1}^{m}r_{i} \wp _{{{{\rho x_{i}}}}}^{\zeta }e^{\phi \wp _{{{{\rho x_{i}}}}}} \right\} \dfrac{ (n-m)!p^{\sum _{ i=0}^{m-1}r_{i}} (1-p)^{ (m-1) (n-m)-\sum _{ i=0}^{m-1} (m-i)r_{i}}}{ \left ( n-m-\sum _{ i=0}^{m-1}r_{i}\right) !\prod _{s=1}^{i-1}r_{s}!} \end{aligned}$$

To derive the *MLE* of model parameters $\Phi$ the natural logarithm of the likelihood function Eq.  (14) becomes

15 $$\begin{aligned} \pounds \left ( x|\Phi \right)&= \log L (x|\Phi )\propto m\log \beta + (m-n_{1})\log \rho + (\zeta -1)\sum _{i=1}^{n_{1}}\log x_{i}+\sum _{i=1}^{n_{1}}\log (\zeta +\phi x_{i})\nonumber \\ &\quad -\beta \sum _{i=1}^{n_{1}} x_{i}^{\zeta }e^{\phi x_{i}}+\phi \sum _{i=1}^{n_{1}} x_{i}-\beta \sum _{i=1}^{n_{1}}r_{i} x_{i}^{\zeta }e^{\phi x_{i}}+ (\zeta -1)\sum _{i=n_{1}+1}^{m} \log \wp _{{{{\rho x_{i}}}}} +\sum _{i=n_{1}+1}^{m}\log (\zeta +\phi \wp _{{{{\rho x_{i}}}}})\nonumber \\ &\quad -\beta \sum _{i=n_{1}+1}^{m} \wp _{{{{\rho x_{i}}}}}^{\zeta }e^{\phi \wp _{{{{\rho x_{i}}}}}}+\phi \sum _{i=n_{1}+1}^{m}\wp _{{{{\rho x_{i}}}}}-\beta \sum _{i=n_{1}+1}^{m}r_{i} \wp _{{{{\rho x_{i}}}}}^{\zeta }e^{\phi \wp _{{{{\rho x_{i}}}}}} \end{aligned}$$

The *MLE* can be derived by solving the gradient function given by Eq.  (A.1) in Appendix. However, since the numerical expression does not offer a closed-form solution, use of gradient-based methods like Newton–Raphson approach or the Quasi-Newton procedure becomes crucial to obtain the *MLE* of $\Phi$. The initial values for the Newton–Raphson method were selected via exploratory analysis of the Alpha Power Weibull Distribution’s theoretical framework. A coarse grid search ($\beta \in [., .]$, $\rho \in [., .]$, $\zeta \in [., .]$, $\phi \in [., .]$) ensured convergence, validated by MLE, MAP, and BAYS estimates.

### Interval estimation

This subsection derives approximate *CI* for the model parameters based on the asymptotic normality of the *MLE*. Let ${\hat{\Phi }}_{i}, i=1,2,3,4$ with $({\hat{\Phi }}_{1}={\hat{\zeta }},{\hat{\Phi }}_{2}={\hat{\phi }},{\hat{\Phi }}_{3}={\hat{\beta }}\ \text {and}\ {\hat{\Phi }}_{4}= {\hat{\rho }})$. The $100 (1 - \alpha )\%$ two-sided approximate *CI* for $\psi _{i}$ takes the form: $\hat{\Phi }_{i} \pm Z_{\alpha /2}\sqrt{Var ({\hat{\Phi }}_{i})}$. Here, $Z_{\alpha /2}$ represents the $(\alpha /2)^{th}$ upper percentile of standard normal distribution.

The log-transformation of the *MLE* given by^31^ can be employed to overcome the performance deficiency of normal distribution when *n* is small. A $100 (1 - \alpha )\%$ two-sided approximate *CI* becoms: $({\hat{\Phi }}_{i}.\exp (-\frac{Z_{\alpha /2}\sqrt{Var ({\hat{\Phi }}_{i})}}{{\hat{\Phi }}_{i}}),{\hat{\Phi }}_{i}.\exp (\frac{Z_{\alpha /2}\sqrt{Var ({\hat{\Phi }}_{i})}}{{\hat{\Phi }}_{i}}))$

## Bayesian estimation

To derive the Bayes estimators of $\Phi$ based on *SSPALT* under $PT\text {-}II CBR$, we consider independent Gamma priors for the unknown parameters as:

$$\begin{aligned} \pi (\zeta )&\propto \zeta ^{a_{1}-1}e^{-\zeta b_{1}}\quad \zeta>0,\qquad \pi (\phi )\propto \phi ^{a_{2}-1}e^{-\phi b_{2}}\quad \phi>0,\\ \pi (\beta )&\propto \beta ^{a_{3}-1}e^{-\beta b_{3}}\quad \beta>0,\qquad \pi (\rho )\propto \rho ^{a_{4}-1}e^{-\rho b_{4}}\quad \rho >1, \end{aligned}$$

where $a_{i} ,\ b_{i}\in (0,\infty )$ for $i=1,2,3,4$ are the hyper parameters. We choose Gamma priors because they encapsulate maximum prior information, as seen in^32^. The joint prior can be written as:

16 $$\begin{aligned} \pi (\Phi )&\propto \zeta ^{a_{1}-1}\phi ^{a_{2}-1}\beta ^{a_{3}-1}\rho ^{a_{4}-1}\exp \left\{ -\zeta b_{1}-\phi b_{2}-\beta b_{3}-\rho b_{4} \right\} . \end{aligned}$$

Now letting $\iota \left ( \Phi \right) = \log \pi (\Phi )$ and since the (transformed) parameters are assumed a priori independent Gamma priors then,

17 $$\begin{aligned} \begin{aligned} \nabla \iota \left ( \Phi \right)&= \begin{pmatrix} \frac{\partial \iota \left ( \Phi \right) }{\partial \zeta } \\ \frac{\partial \iota \left ( \Phi \right) }{\partial \phi } \\ \frac{\partial \iota \left ( \Phi \right) }{\partial \beta } \\ \frac{\partial \iota \left ( \Phi \right) }{\partial \rho } \end{pmatrix}= \begin{pmatrix} \dfrac{a_{1}-1}{ \zeta }- b_{1}\\ \dfrac{a_{2}-1}{\phi }- b_{2}\\ \dfrac{a_{3}-1}{ \beta }- b_{3}\\ \dfrac{a_{4}-1}{\rho }- b_{4} \end{pmatrix} \end{aligned}, \end{aligned}$$

The hyperparameters of the priors can be determined in practice from the available past data. For example, lets there are *Z* past available observations from *MWD*. For each sample, we can compute the *MLE* (${\hat{\zeta }}_u$, ${\hat{\phi }}_u$, ${\hat{\beta }}_u$, ${\hat{\rho }}_u$) for $u = 1, 2, \ldots , U$. By using the mean and variance of *MLE* with that of the gamma distribution, one can derive the hyperparameter values. For instance, for $\zeta$, where the mean and variance of $\zeta$ are $a_1/b_1$ and $a_1/b_1^2$, respectively, we obtain:

$$\begin{aligned} a_1&= \frac{\left[ \sum _{u=1}^{U} {\hat{\zeta }}_u /U\right] ^2}{\sum _{u=1}^{U-1} \left[ {\hat{\zeta }}_u - \sum _{u=1}^{U} {\hat{\zeta }}_u / U\right] ^2 / (U-1)}, \\ b_1&= \frac{\sum _{u=1}^{U} {\hat{\zeta }}_u / U}{\sum _{u=1}^{U-1} \left[ {\hat{\zeta }}_u - \sum _{u=1}^{U} {\hat{\zeta }}_u / U\right] ^2 / (U-1)}. \end{aligned}$$

Similarly, we can obtain hyperparameter values for the prior densities of $\phi$, $\beta$, and $\rho$. Additionally, the joint posterior function combines the likelihood of Eq.  (14) with Eq.  (16) via Bayes theorem as:

18 $$\begin{aligned} \eth (\Phi |{{\textbf {X}}})&= K^{-1} \zeta ^{a_{1}-1}\phi ^{a_{2}-1}\beta ^{m+a_{3}-1}\rho ^{m-n_{1}+a_{4}-1} \exp \left\{ - \zeta b_{1}-\phi b_{2}-\beta b_{3}-\rho b_{4} \right\} \nonumber \\ &\quad \times \prod _{i=1}^{n_{1}} x_{i}^{\zeta -1} (\zeta +\phi x_{i}) \exp \left\{ -\sum _{i=1}^{n_{1}}\left ( \beta (r_{i}+1) x_{i}^{\zeta }e^{\phi x_{i}}-\phi x_{i}\right) \right\} \nonumber \\ &\quad \times \prod _{i=n_{1}+1}^{m} \wp _{{{{\rho x_{i}}}}} ^{\zeta -1}\left ( \zeta +\phi \wp _{{{{\rho x_{i}}}}}\right) \exp \left\{ -\sum _{i=n_{1}+1}^{m}\left ( \beta (r_{i}+1) \wp _{{{{\rho x_{i}}}}}^{\zeta }e^{\phi \wp _{{{{\rho x_{i}}}}}}-\phi \wp _{{{{\rho x_{i}}}}}\right) \right\} \end{aligned}$$

### The maximum a posteriori (*MAP*) estimation

In this part of the study, the *MAP* approach, also known as generalized *MLE* (*GMLE*), is considered. *MAP* estimation aims to maximize the posterior density rather than the likelihood function.

The result in the maximum a posteriori framework is equivalent to the result using *MLE* but with an added regularization term. This regularization term is nothing else but the prior of the weights. Thus, the estimates of the parameters, obtained using the log of the posterior density Eq.  (18), via *MAP* can be formulated as follows:

19 $$\begin{aligned} {\hat{\Phi }}_{MAP}&= \arg \max \nabla _{\Phi }\log \eth (\Phi |{{\textbf {X}}}) = \arg \max _{\Phi }\left[ \pounds \left ( x|\Phi \right) +\iota (\Phi )\right] \nonumber \\&= \begin{pmatrix} \frac{\partial \log \eth (\Phi |{{\textbf {X}}}) }{\partial \zeta } \\ \frac{\partial \log \eth (\Phi |{{\textbf {X}}}) }{\partial \phi } \\ \frac{\partial \log \eth (\Phi |{{\textbf {X}}}) }{\partial \beta } \\ \frac{\partial \log \eth (\Phi |{{\textbf {X}}}) }{\partial \rho } \end{pmatrix} = \begin{pmatrix} \frac{\partial \pounds (x|\Phi ) }{\partial \zeta }+\dfrac{a_{1}-1}{\zeta } + b_{1} \\ \frac{\partial \pounds (x|\Phi ) }{\partial \phi }+\dfrac{a_{2}-1}{\zeta } + b_{2} \\ \frac{\partial \pounds (x|\Phi ) }{\partial \beta }+\dfrac{m+a_{3}-1}{\beta } + b_{3} \\ \frac{\partial \pounds (x|\Phi ) }{\partial \rho }+\dfrac{m-n_{1}+a_{4}-1}{\zeta } + b_{4} \end{pmatrix}, \end{aligned}$$

The *MAP* estimates are derived by solving these equations relative to $\Phi$. However, as the numerical expression does not offer a closed-form solution, numerical methods are used to obtain the estimates. Besides, the confidence intervals for the parameters using the MAP method are constructed similarly to the MLE method (section “Interval estimation”), with the key difference being the incorporation of prior information. The MLE are replaced with the MAP estimates and the variances for confidence intervals are computed using the sum of the diagonal elements of the total Fisher information matrix. The total Fisher information matrix for MAP estimates is obtained by summing the Fisher information of the MLE (Eq. A.2) and the Fisher information from the joint prior (Eq. A.3).

### Deriving Bayes estimators with different loss functions

This section explores the derivation of Bayes estimators under various loss functions. We will consider three specific functions: *SELF*, *LINEX*, and the *GELF*. The symmetric *SELF* treats overestimation and underestimation equally. However, in practical applications, overestimating or underestimating a parameter can have significantly different consequences. For situations where overestimation or underestimation has a more significant impact, asymmetrical loss functions, *LINEX* and *GELF*, offer a more suitable approach. These functions assign greater weight to one type of error compared to the other. The *LINEX* can be written as:

$$\begin{aligned} L_{ln} ({\hat{\Phi }}, \Phi ) = e^{k_{1} ({\hat{\Phi }} - \Phi )} - k_{1} ({\hat{\Phi }} - \Phi ) - 1, \end{aligned}$$

Since this is a convex loss function, its shape is decided by its loss parameter, denoted by $k_{1}$. The estimator under LINEX is:

20 $$\begin{aligned} {\hat{\Phi }}_{ln} =-\frac{1}{k_{1}} \log \left[ E_{p}\left ( e^{-k_{1}\Phi } \right) \right] . \end{aligned}$$

The *GELF* is written as:

$$\begin{aligned} L_{ge} ({\hat{\Phi }}, \Phi ) \propto \left ( \frac{{\hat{\Phi }}}{\Phi }\right) ^{k_{2}} - k_{2}\log \left ( \frac{{\hat{\Phi }}}{\Phi }\right) - 1 \end{aligned}$$,

And the corresponding Bayesian estimator is

21 $$\begin{aligned} {\hat{\Phi }}_{ge} = \left[ E_{p}\left ( \Phi ^{-k_{2}}\right) \right] ^{-\frac{1}{k_{2}}},\end{aligned}$$

where $k_{2}$ is a shape parameter that reflects departure from symmetry. Next, we discuss the estimation under balanced loss functions (see^33^) defined as

22 $$\begin{aligned} L_{b } ({\hat{\Phi }},\Phi )\propto \varepsilon \ L (\Phi _{0},\Phi )+ (1-\varepsilon )\ L ({\hat{\Phi }},\Phi ) \end{aligned}$$

where $\ L$ denotes some arbitrary loss, $\Phi _{0}$ is the target estimator for $\Phi$, say *MLE* and $\varepsilon \in (0, 1)$ is the weight. In Eq.  (22), the choice $\ L (\Phi _{0},\Phi )= \left ( {\hat{\Phi }}-\Phi \right) ^{2}$ leads to balanced squared error loss (BSEL) function. Bayes estimate of $\Phi$ under *BSEL* is given by

23 $$\begin{aligned} {\hat{\Phi }}_{B bse} = \varepsilon \Phi _{0} + (1-\varepsilon ) E_{p}\left ( \Phi \right) . \end{aligned}$$

Notice that by considering $\ L ({\hat{\Phi }},\Phi ) =L_{ln} ({\hat{\Phi }},\Phi ),$ in Eq.  (22) we obtain the balanced *LINEX* (*BLINEX*) loss function. The corresponding estimator is of the form

24 $$\begin{aligned} {\hat{\Phi }}_{B bln} =-\frac{1}{k_{1}} \log \left[ \varepsilon e^{-k_{1}\Phi _{0}} + (1-\varepsilon ) E_{p}\left ( e^{-k_{1}\Phi } \right) \right] . \end{aligned}$$

Similarly, the estimator of $\Phi$ relative to balanced *GELF* (*BGELF*) is given by

25 $$\begin{aligned} {\hat{\Phi }}_{B bge} = \left[ \varepsilon \Phi _{0}^{-k_{2}} + (1-\varepsilon )E_{p}\left ( \Phi ^{-k_{2}} \right) \right] ^{-\frac{1}{k_{2}}}. \end{aligned}$$

As the estimators derived under the proposed loss functions (Eqs. (20)– (25)) involve ratios having integrals in the denominator. These expressions lack closed-form solutions, making direct evaluation of the posterior expectation computationally expensive. Therefore, various Markov Chain Monte Carlo (*MCMC*) techniques are employed to approximate these integrals, enabling the practical computation of the Bayes estimators.

#### Metropolis-Hastings algorithm

Due to the complex nature of the joint posterior density given by Eq. (18), direct data generation is not feasible. Therefore, this subsection focuses on deriving the full conditional posterior distributions for the components of the parameter vector $\vec {\varPsi }$ using MCMC techniques, specifically the Gibbs sampler and the Metropolis-Hastings (MH) algorithm. These full conditional distributions are crucial for implementing these MCMC methods to obtaining Bayes estimates. The conditional posterior distributions are derived as follows:

26 $$\begin{aligned} \eth (\rho |\zeta ,\phi ,\beta ,{{\textbf {Y}}}) &\propto \rho ^{m-n_{1}+a_{4}-1} \prod _{i=n_{1}+1}^{m} \wp _{{{{\rho x_{i}}}}} ^{\zeta -1}\left ( \zeta +\phi \wp _{{{{\rho x_{i}}}}}\right) \nonumber \\ &\quad \times \exp \left\{ -\sum _{i=n_{1}+1}^{m}\left ( \beta (r_{i}+1) \wp _{{{{\rho x_{i}}}}}^{\zeta }e^{\phi \wp _{{{{\rho x_{i}}}}}}-\phi \wp _{{{{\rho x_{i}}}}}\right) -\rho b_{4}\right\} , \end{aligned}$$

27 $$\begin{aligned} \eth (\zeta |\phi ,\beta ,\rho ,{{\textbf {Y}}}) &\propto \zeta ^{a_{1}-1}\prod _{i=1}^{n_{1}} x_{i}^{\zeta -1} (\zeta +\phi x_{i}) \prod _{i=n_{1}+1}^{m} \wp _{{{{\rho x_{i}}}}} ^{\zeta -1}\left ( \zeta +\phi \wp _{{{{\rho x_{i}}}}}\right) \nonumber \\ &\quad \times \exp \left\{ -\beta \sum _{i=1}^{n_{1}} (r_{i}+1) x_{i}^{\zeta }e^{\phi x_{i}} -\beta \sum _{i=n_{1}+1}^{m} (r_{i}+1) \wp _{{{{\rho x_{i}}}}}^{\zeta }e^{\phi \wp _{{{{\rho x_{i}}}}}}-\zeta b_{1}\right\} , \end{aligned}$$

28 $$\begin{aligned} \eth (\phi |\zeta ,\beta ,\rho ,{{\textbf {Y}}}) &\propto \phi ^{a_{2}-1} \prod _{i=1}^{n_{1}} (\zeta +\phi x_{i}) \exp \left\{ -\sum _{i=1}^{n_{1}}\left ( \beta (r_{i}+1) x_{i}^{\zeta }e^{\phi x_{i}}-\phi x_{i}\right) - \phi b_{2} \right\} \nonumber \\ &\quad \times \prod _{i=n_{1}+1}^{m} \left ( \zeta +\phi \wp _{{{{\rho x_{i}}}}}\right) \exp \left\{ -\sum _{i=n_{1}+1}^{m}\left ( \beta (r_{i}+1) \wp _{{{{\rho x_{i}}}}}^{\zeta }e^{\phi \wp _{{{{\rho x_{i}}}}}}-\phi \wp _{{{{\rho x_{i}}}}}\right) \right\} , \end{aligned}$$

29 $$\begin{aligned} \eth (\beta |\zeta ,\phi ,\rho ,{{\textbf {Y}}})&\propto \beta ^{m+a_{3}-1} \exp \left\{ -\beta \left ( \sum _{i=1}^{n_{1}} (r_{i}+1) x_{i}^{\zeta }e^{\phi x_{i}}+\sum _{i=n_{1}+1}^{m} (r_{i}+1) \wp _{{{{\rho x_{i}}}}}^{\zeta }e^{\phi \wp _{{{{\rho x_{i}}}}}}+ b_{3}\right) \right\} . \end{aligned}$$

It’s evident that the density of $\beta$ in Eq. (29) follows a gamma distribution. Therefore, generating $\beta$ can be easily accomplished using any generation routine. However, the conditionals of $\rho$, $\zeta$, and $\phi$ in Eqs. (26–28) do not conform to any well-known standard forms. As a result, Gibbs sampling is not an appropriate option for these parameters, necessitating the use of a *MH* sampler for the implementation of the *MCMC* methodology.

**Fig. 1:**
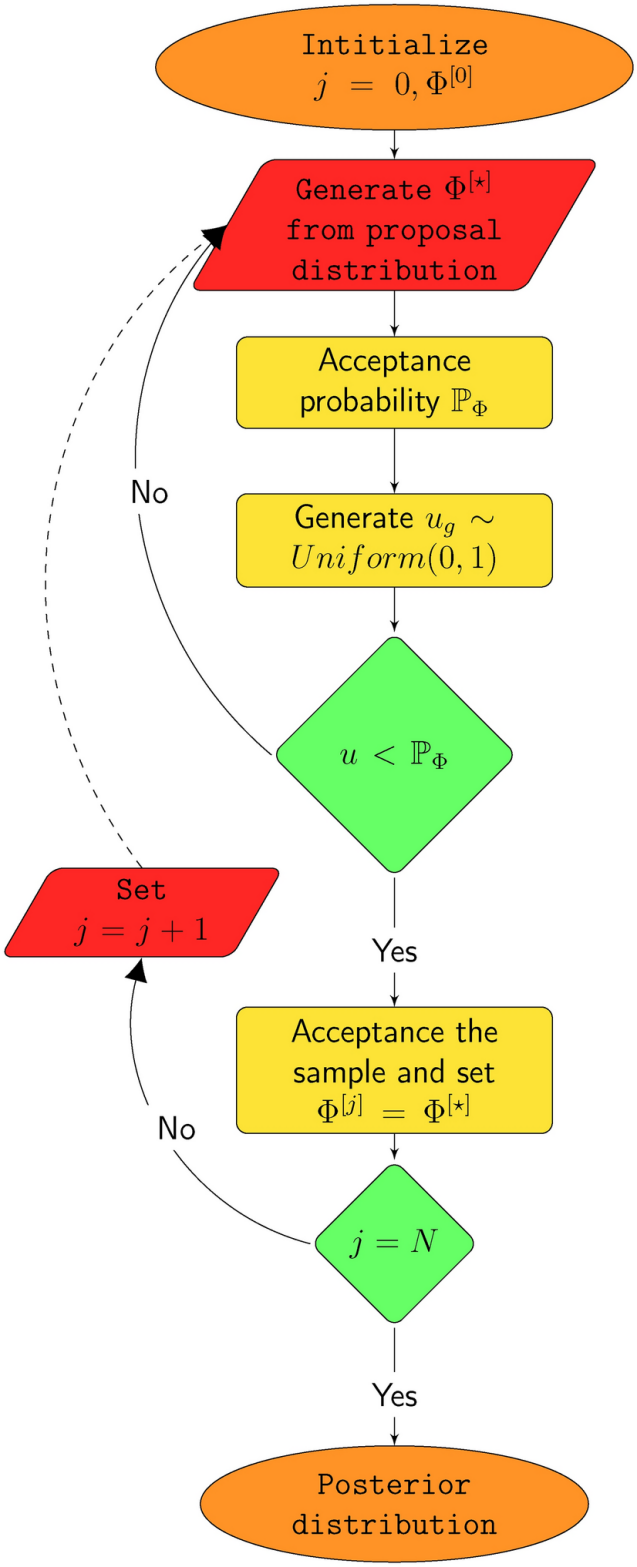
Major steps of *MH* algorithm.

Hence, we describe the *MH* Algorithm, which is a crucial component in generating posterior samples using a hybrid algorithm combining Gibbs sampling and *MH* steps for Bayes estimation. The flowchart depicted by Fig. 1 offers the working of *MH* procedure. Given the full conditionals described in Eqs.  (26–29), we outline the following steps to effectively utilize the *MH* Algorithm within the Gibbs sampling technique for estimating the parameters $\rho , \zeta$, and $\phi$ (see e.g.^34^):

**Algorithm 1:**
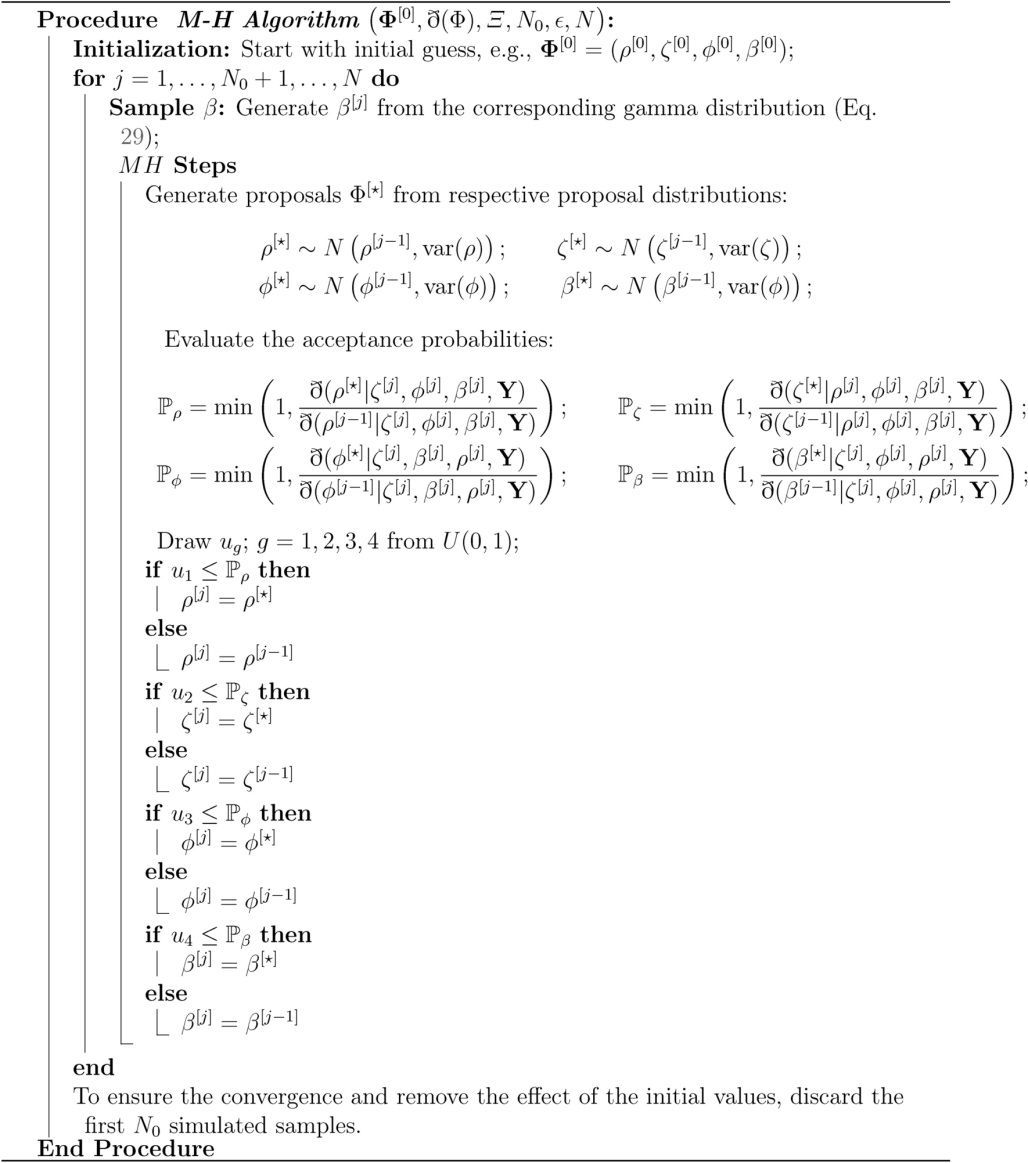
Metropolis-Hastings (*MH*) algorithm.

By following these steps, we can effectively generate samples using the *MH* Algorithm within the Gibbs sampling for Bayes estimation. Then the Bayes estimates of $\Phi$ relative to *SELF*, *LINEX* and *GELF* are obtained using the approximate posterior samples, $\Phi ^{[l]},$ produced under algorithm (1) and Fig. 1, as:

30 $$\begin{aligned} {\hat{\Phi }}_{se}= \dfrac{\sum _{l=N_{0}+1}^{N} \Phi ^{[l]} }{N-N_{0}},\end{aligned}$$

31 $$\begin{aligned} {\hat{\Phi }}_{ln}= -\frac{1}{k_{1}} \log \left[ \dfrac{1 }{ N-N_{0} } \sum _{l=N_{0}+1}^{N}e^{k_{1} \Phi ^{[l]} } \right] , \end{aligned}$$

32 $$\begin{aligned} {\hat{\Phi }}_{ge}= \left ( \dfrac{\sum _{l=N_{0}+1}^{N} (\sum _{l=N_{0}+1}^{N} \Phi ^{[l]} )^{-k_{2}} }{N-N_{0}}\right) ^{-\frac{1}{k_{2}}}. \end{aligned}$$

To calculate the Highest Posterior Density (*HPD*) *CI* of $\Phi$ we take the quantiles as the limits for intervals^35^. Sort $\Phi ^{[N_{0}+1]}, \Phi ^{[N_{0}+2]},\dots , \Phi ^{[N]}$ as $\Phi ^{ (1)}, \Phi ^{ (2)},\ldots , \Phi ^{ (N-N_{0})}$. Hence the $100 (1 - \alpha )\%$ *HPD* for $\Phi$ is

$$\begin{aligned} \left\{ \Phi _{\big [ (N-N_{0}) (\alpha /2)\big ]},\Phi _{\big [ (N-N_{0}) (1-\alpha /2)\big ]}\right\} . \end{aligned}$$

## Numerical evaluation

This part of study evaluates the applicability of the considered estimation procedure vis a simulation study and real example. We compare the efficiency, accuracy, and convergence properties of the approaches discussed in previous sections. We consider different scenarios and parameter settings to assess the robustness and effectiveness of each method. For the simulation study, we generate data according to specific distribution and model assumptions. We then apply the various estimation methods discussed earlier to estimate the parameters based on the simulated samples. Performance metrics such as mean square error (MSE), and convergence rates are used to evaluate and compare the methods. Additionally, we present a real-world example where we apply the proposed Bayesian estimation techniques to analyze real data. This example demonstrates the practical applicability of the methods in solving real problems and making meaningful inferences from data. Overall, the numerical evaluation aims to provide insights into the strengths and limitations of each method, helping researchers and practitioners choose the most suitable approach for their specific modeling and estimation tasks.

### Simulation findings

Since our suggested techniques do not allow for an analytical study of estimator behaviour, here we give numerical examples based on a simulation-based approach. We take into account diverse *SSPALT* under $PT\text {-}II CBR$ samples with distinct binomial elimination patterns of observations during testing. We use the algorithm described by^26^ for simulation. Analysing the consequences of varying these parameters in different combinations may help identify an acceptable censorship method for practical issues.

We compare several estimators based on *SSPALT* under $PT\text {-}II CBRs$ that were obtained in sections “Likelihood estimation of model parameters” and “Bayesian estimation”. The simulated Expected Estimation Loss (EEL) used in this comparison are based on the average loss over the sample for all considered unbalanced and balanced loss functions. It is important to note that because the estimators are not in closed form, precise expressions for the EEL cannot be determined. As such, the estimators’ EEL are calculated using *MC* simulation experiment with 7000 samples. It should also be noted that a number of parameters, such as $n, m, p, \varrho _1,$ and $\varrho _2$ have an impact on the estimators’ EEL. We have calculated the simulated EEL for the following scenarios in order to examine the effects of different combinations of the total sample size (*n*) with various values of the effective sample size (*m*):

i with *n* = 100 and *m* = 40,
ii with *n* = 100 and *m* = 30,
iii with *n* = 100 and *m* = 20,
iv with *n* = 200 and *m* = 80,
v with *n* = 200 and *m* = 50,
vi with *n* = 200 and *m* = 30.

Different combinations of stress factors $\varrho _1 = 1.5,\varrho _2 = 6.0$ and $\varrho _1 = 0.8,\varrho _2 = 5.5$ are applied to each scenario, and a probability of removals (*p*) of 0.4 is used for evaluation. For asymmetric loss functions, we have taken into consideration two values for the loss parameters ($k_{1}=k_{2}$)= 0.5, which states that the overestimation is more consequential than that of the underestimation, and -0.5, which indicates that underestimation is more serious than overestimation. Besides, for the considered balanced loss functions the value of weight parameter was set at $\varepsilon =0.5$. Simulated *MSE* under *SELF*, *LINEX*, *GELF*, *BLINEX*, and *BGELF* have been computed for selected values of these parameters after *SSPALT* under $PT\text {-}II CBR$. Samples were generated using the *MH* algorithm described in section “Bayesian estimation”.

#### Discussion of results

The results for $\varrho _1$ = 1.5, $\varrho _2$ = 6, $\rho$ = 2, $\beta$ = 0.1, $\zeta$ = 0.3, $\phi$ = 0.5, *p* = 0.4; and $\varrho _1$ = 1.5, $\varrho _2$ = 6, $\rho$ = 2, $\beta$ = 0.1, $\zeta$ = 0.3, $\phi$ = 0.5, *p* = 0.4 for the loss parameters ($k_{1}=k_{2}$= 0.5, -0.5) and using the weight parameter as $\varepsilon =0.5$ are presented in Tables 1, 2; and Tables 3, 4, respectively. The corresponding results for coverage probability and average length of the *HPD* and *CI* for the estimators are shown in Table 5. By looking at Tables 1 2, 3, 4 we can see that as the effective sample size *m* reduces, the EEL connected to each estimator of $\zeta , \phi , \beta$ and $\rho$ grow. Notably, among all estimation methods and loss functions, the hazards associated with the suggested estimators of $\zeta , \beta$ and $\rho$ under *BGELF* are found to be lowest when $k_{2} < 0$ (-0.5 in our case) as well as $k_{2} > 0$, followed by *GELF*. However, in the case of $\phi$ it is found that the EEL associated with the suggested estimators under *GELF* are the lowest, followed by *BGELF*, for most of the considered scenarios (see e.g. Table 2 and 4).However, given the studied values of *n* and *m*, the EEL for the Bayes estimators were consistently found to be the lowest for all estimators of $\zeta , \phi , \beta$ and $\rho$, regardless of whether the associated loss parameters $k_{1},k_{2} < 0$ or $k_{1},k_{2} > 0$. It’s also important to note that, for both $k_{2} < 0$ or $k_{2} > 0$ under *BGELF* and *GELF*, the total risk magnitudes consistently showed lower dimensions for $k_{2} > 0$.

We analyze the behavior of the average interval length (*AL*) and coverage probability (*CP*) across various parameter combinations, as presented in Table 5, which provides interval estimates using both the classical confidence interval (*CI*) and the highest posterior density (*HPD*) interval. A consistent trend is observed: the average interval length decreases as the number of failures *m* increases, reflecting an improvement in estimation precision as more failure data become available. Among all the estimation approaches considered, the Bayesian estimators?particularly those based on the *HPD* intervals?demonstrate the shortest average interval length, indicating their efficiency in parameter estimation. The MAP-based *CI* follows closely in performance, while traditional frequentist confidence intervals tend to produce wider intervals, suggesting a higher level of uncertainty in parameter estimation. Furthermore, the *CP* exhibits a decreasing trend with increasing *m* across most of the examined scenarios, when the total sample size *n* remains fixed. This reduction in *CP* is expected, as larger *m* values reduce the available censored observations, thereby impacting interval estimation accuracy. Despite this, the coverage probabilities of the Bayesian *HPD* estimators remain consistently high, exceeding 90% for all tested values of *n* and *m*. This robustness underscores the advantage of Bayesian inference in ensuring reliable interval estimates, reinforcing the superiority of the Bayesian approach over conventional methods.

**Table 1:** Estimates (Risk of estimates) under *MLE*, *MAP*, *SELF* and *BSELF* with fixed value, $\varrho _1$ = 1.5, $\varrho _2$ = 6, $\rho$ = 2, $\beta$ = 0.1, $\zeta$ = 0.3, $\phi$ = 0.5, *p* = 0.4, $\varepsilon =0.5$.

(n,m)	Method	${\hat{\zeta }}$	${\hat{\phi }}$	${\hat{\beta }}$	${\hat{\rho }}$
(100,40)	*MLE*	0.4005 (0.1336)	0.436 (0.1694)	0.1072 (0.0317)	1.4756 (1.8281)
	*MAP*	0.567 (0.1043)	0.3441 (0.1343)	0.1129 (0.0312)	1.3749 (0.5524)
	*SELF*	0.5503 (0.0662)	0.7610 (0.1133)	0.2443 (0.0268)	2.0582 (0.0247)
	*BSELF*	0.5106 (0.0465)	0.7617 (0.5390)	0.2144 (0.0166)	2.0128 (0.0248)
(100,30)	*MLE*	0.4055 (0.1367)	0.4265 (0.1758)	0.1098 (0.12)	1.5653 (8.0378)
	*MAP*	0.5835 (0.1178)	0.3661 (0.0279)	0.1 (0.087)	1.5522 (0.4255)
	*SELF*	0.6095 (0.1027)	0.7979 (0.1172)	0.3344 (0.0744)	2.1203 (0.0564)
	*BSELF*	0.5598 (0.0724)	0.9365 (1.2974)	0.2822 (0.0461)	2.0842 (0.0276)
(100,20)	*MLE*	0.4227 (0.1509)	0.3956 (0.1889)	0.0977 (0.2114)	2.8523 (0.09564)
	*MAP*	0.8399 (0.5044)	0.4284 (0.153)	0.0566 (0.21)	1.7071 (0.3419)
	*SELF*	0.6424 (0.1218)	0.8015 (0.1193)	0.476 (0.1641)	2.1221 (0.0574)
	*BSELF*	0.6018 (0.0943)	2.0499 (22.4623)	0.4354 (1.0123)	2.0855 (0.0282)
(200,80)	*MLE*	0.3846 (0.1164)	0.4322 (0.1397)	0.1074 (0.011)	1.2635 (0.9326)
	*MAP*	0.4537 (0.0314)	0.3263 (0.0417)	0.1164 (0.0008)	1.5227 (0.4639)
	*SELF*	0.4089 (0.014)	0.6756 (0.0525)	0.1081 (0.0004)	2.1056 (0.0477)
	*BSELF*	0.4045 (0.0126)	0.5648 (0.0271)	0.1102 (0.0004)	2.0739 (0.0234)
(200,50)	*MLE*	0.3881 (0.1173)	0.4247 (0.1447)	0.1088 (0.0111)	1.3483 (1.8124)
	*MAP*	0.4503 (0.0302)	0.3443 (0.1346)	0.1049 (0.0094)	2.0202 (0.4532)
	*SELF*	0.4838 (0.0388)	0.7633 (0.0959)	0.1571 (0.0062)	2.1104 (0.0523)
	*BSELF*	0.4595 (0.0291)	0.645 (0.0817)	0.1466 (0.0042)	2.0772 (0.0256)
(200,30)	*MLE*	0.4004 (0.1263)	0.3746 (0.1667)	0.0806 (0.111)	3.1576 (1.9324)
	*MAP*	0.4501 (0.0292)	0.3613 (0.1313)	0.1004 (0.092)	2.5203 (0.4423)
	*SELF*	0.6095 (0.1027)	0.8011 (0.1188)	0.3366 (0.0761)	2.1218 (0.057)
	*BSELF*	0.5602 (0.0726)	0.9477 (1.3862)	0.2842 (0.0473)	2.0853 (0.0279)

**Table 2:** Estimates (Risk of estimates) under *LINEX*, *GELF*, *BLINEX* and *BGELF* with fixed values of, $\varrho _1$ = 1.5, $\varrho _1$ = 6, $\rho$ = 2, $\beta$ = 0.1, $\zeta$ = 0.3, $\phi$ = 0.5, *p* = 0.4, $\varepsilon =0.5$.

(n,m)	Method	$k_{1}=k_{2}=-0.5$				$k_{1}=k_{2}=0.5$			
		${\hat{\zeta }}$	${\hat{\phi }}$	${\hat{\beta }}$	${\hat{\rho }}$	${\hat{\zeta }}$	${\hat{\phi }}$	${\hat{\beta }}$	l
(100,40)	*LINEX*	0.5516 (0.0669)	0.7679 (0.1184)	0.2454 (0.0273)	2.0615 (0.0252)	0.5490 (0.0655)	0.7542 (0.1083)	0.2432 (0.0264)	2.0548 (0.0242)
	*GELF*	0.5478 (0.0650)	0.7525 (0.1076)	0.2404 (0.0253)	2.0565 (0.0245)	0.5424 (0.0624)	0.7348 (0.0963)	0.2323 (0.0223)	2.0533 (0.0241)
	*BLINEX*	0.5127 (0.0474)	1.0103 (2.0205)	0.2160 (0.0171)	2.0196 (0.0231)	0.5085 (0.0455)	0.6065 (0.1234)	0.2128 (0.0160)	2.0056 (0.0270)
	*BGELF*	0.5066 (0.0447)	0.5580 (0.2856)	0.2081 (0.0147)	2.0087 (0.0264)	0.4983 (0.0413)	0.1608 (0.2676)	0.1958 (0.0114)	2.0000 (0.0303)
(100,30)	*LINEX*	0.6111 (0.1038)	0.8041 (0.1218)	0.3372 (0.0765)	2.1246 (0.0581)	0.6079 (0.1017)	0.7917 (0.1126)	0.3316 (0.0724)	2.1161 (0.0548)
	*GELF*	0.6069 (0.1011)	0.7902 (0.1121)	0.3271 (0.07)	2.1184 (0.0557)	0.6015 (0.0977)	0.7746 (0.1022)	0.312 (0.0613)	2.1145 (0.0545)
	*BLINEX*	0.5626 (0.074)	1.3778 (8.5275)	0.2864 (0.0485)	2.0902 (0.0298)	0.557 (0.0708)	0.6931 (0.1595)	0.278 (0.0438)	2.0783 (0.0257)
	*BGELF*	0.5548 (0.0697)	0.6896 (0.4968)	0.2697 (0.0402)	2.0815 (0.0268)	0.5448 (0.0644)	0.2691 (0.2797)	0.245 (0.03)	2.076 (0.0252)
(100,20)	*LINEX*	0.644 (0.123)	0.8079 (0.1241)	0.4822 (0.17)	2.1264 (0.0591)	0.6407 (0.1206)	0.7951 (0.1145)	0.4698 (0.1584)	2.1179 (0.0558)
	*GELF*	0.6398 (0.12)	0.7937 (0.114)	0.4633 (0.1535)	2.1202 (0.0568)	0.6344 (0.1163)	0.7777 (0.1037)	0.436 (0.1321)	2.1162 (0.0555)
	*BLINEX*	0.6045 (0.096)	1.4778 (11.623)	0.4803 (9.5537)	2.0915 (0.0303)	0.5991 (0.0926)	0.7289 (0.2149)	0.4103 (0.1201)	2.0795 (0.0262)
	*BGELF*	0.5973 (0.0916)	1.1844 (4.6588)	0.4046 (0.2442)	2.0827 (0.0273)	0.5881 (0.0861)	0.2926 (0.3396)	0.3565 (0.0867)	2.0772 (0.0257)
(200,80)	*LINEX*	0.4095 (0.0141)	0.6788 (0.054)	0.1082 (0.0004)	2.109 (0.0489)	0.4084 (0.0138)	0.6724 (0.051)	0.1081 (0.0004)	2.1022 (0.0466)
	*GELF*	0.4076 (0.0137)	0.6709 (0.0507)	0.1077 (0.0004)	2.104 (0.0472)	0.4049 (0.0131)	0.6612 (0.0472)	0.1068 (0.0004)	2.1008 (0.0463)
	*BLINEX*	0.4049 (0.0127)	0.5789 (0.0292)	0.1102 (0.0004)	2.0788 (0.0249)	0.404 (0.0125)	0.55 (0.0256)	0.1101 (0.0004)	2.069 (0.0219)
	*BGELF*	0.4033 (0.0124)	0.5104 (0.0325)	0.1097 (0.0004)	2.0716 (0.0228)	0.4011 (0.0119)	0.3846 (0.0935)	0.1088 (0.0004)	2.067 (0.0216)
(200,50)	*LINEX*	0.4847 (0.0392)	0.7683 (0.0993)	0.1574 (0.0063)	2.1142 (0.0537)	0.4829 (0.0384)	0.7583 (0.0926)	0.1568 (0.0062)	2.1065 (0.0509)
	*GELF*	0.4819 (0.0381)	0.7568 (0.0921)	0.1557 (0.006)	2.1086 (0.0517)	0.4781 (0.0367)	0.7436 (0.0847)	0.1528 (0.0055)	2.1049 (0.0507)
	*BLINEX*	0.4606 (0.0295)	0.6846 (0.1301)	0.1469 (0.0043)	2.0827 (0.0275)	0.4584 (0.0287)	0.6075 (0.0581)	0.1463 (0.0042)	2.0718 (0.0239)
	*BGELF*	0.4572 (0.0283)	0.5294 (0.0709)	0.1449 (0.0039)	2.0747 (0.0249)	0.4527 (0.0267)	0.2726 (0.193)	0.1415 (0.0034)	2.0696 (0.0235)
(200,30)	*LINEX*	0.6111 (0.1037)	0.8074 (0.1235)	0.3394 (0.0783)	2.1261 (0.0586)	0.6079 (0.1016)	0.7949 (0.1142)	0.3337 (0.0741)	2.1176 (0.0554)
	*GELF*	0.6069 (0.101)	0.7934 (0.1137)	0.3292 (0.0716)	2.1198 (0.0563)	0.6015 (0.0977)	0.7777 (0.1036)	0.3139 (0.0626)	2.1159 (0.0551)
	*BLINEX*	0.563 (0.0742)	1.4201 (9.2071)	0.2884 (0.0499)	2.0913 (0.03)	0.5574 (0.071)	0.6905 (0.1578)	0.28 (0.0449)	2.0793 (0.0259)
	*BGELF*	0.5553 (0.0699)	0.692 (0.5256)	0.2716 (0.0413)	2.0825 (0.0271)	0.5455 (0.0647)	0.2605 (0.2827)	0.2469 (0.0309)	2.077 (0.0255)

**Table 3:** Estimates (Risk of estimates) under *MLE*, *MAP*, *SELF* and *BSELF* with fixed value of, $\varrho _1$ = 0.8, $\varrho _2$ = 5.5, $\rho$ = 2, $\beta$ = 0.1, $\zeta$ = 0.3, $\phi$ = 0.5, *p* = 0.4, $\varepsilon =0.5$.

(n,m)	Method	${\hat{\zeta }}$	${\hat{\phi }}$	${\hat{\beta }}$	${\hat{\rho }}$
(100,40)	*MLE*	0.3864 (0.1328)	0.4885 (0.3455)	0.0997 (0.0514)	1.687 (5.1544)
	*MAP*	0.6604 (0.2889)	0.5925 (0.1449)	0.1043 (0.049)	0.7881 (1.5391)
	*SELF*	0.5435 (0.0664)	0.782 (0.1077)	0.2188 (0.023)	2.124 (0.0575)
	*BSELF*	0.5059 (0.0473)	0.719 (0.2227)	0.1929 (0.0144)	2.087 (0.0285)
(100,30)	*MLE*	0.3881 (0.3631)	0.4925 (0.1547)	0.1011 (0.1162)	1.7182 (8.1025)
	*MAP*	0.6669 (0.3043)	0.6205 (0.1533)	0.099 (0.088)	0.8353 (1.4423)
	*SELF*	0.6108 (0.1035)	0.8032 (0.1208)	0.3367 (0.0755)	2.1176 (0.0556)
	*BSELF*	0.5616 (0.0734)	0.9468 (1.3553)	0.2846 (0.0471)	2.0823 (0.0273)
(100,20)	*MLE*	0.398 (0.392)	0.4683 (0.178)	0.1052 (0.2213)	1.8113 (6.012)
	*MAP*	0.7516 (0.5032)	0.6953 (0.1698)	0.0816 (0.1824)	0.9983 (1.1488)
	*SELF*	0.6402 (0.1203)	0.8082 (0.1236)	0.4765 (0.1648)	2.1209 (0.058)
	*BSELF*	0.6005 (0.0936)	2.0591 (25.1588)	0.4222 (0.1362)	2.0846 (0.0284)
(200,80)	*MLE*	0.3703 (0.1313)	0.4703 (0.1762)	0.1006 (0.1117)	1.337 (1.2135)
	*MAP*	0.4515 (0.0334)	0.4455 (0.1264)	0.1057 (0.095)	1.1088 (0.9158)
	*SELF*	0.6108 (0.1035)	0.8032 (0.1208)	0.3367 (0.0755)	2.1176 (0.0556)
	*BSELF*	0.5616 (0.0734)	0.9468 (1.3553)	0.2846 (0.0471)	2.0823 (0.0273)
(200,50)	*MLE*	0.3758 (0.1148)	0.4519 (0.1908)	0.1045 (0.111)	1.3817 (1.2466)
	*MAP*	0.446 (0.0317)	0.4833 (0.1246)	0.1 (0.0015)	1.1614 (0.8437)
	*SELF*	0.4853 (0.0393)	0.7633 (0.0954)	0.1586 (0.0065)	2.116 (0.0547)
	*BSELF*	0.4607 (0.0294)	0.6473 (0.0849)	0.1478 (0.0044)	2.0811 (0.0268)
(200,30)	*MLE*	0.3844 (0.1173)	0.4124 (0.1183)	0.1053 (0.1111)	1.9321 (1.8692)
	*MAP*	0.474 (0.0441)	0.5583 (0.1175)	0.0778 (0.097)	1.55 (0.4584)
	*SELF*	0.6102 (0.103)	0.802 (0.1197)	0.3345 (0.0742)	2.1205 (0.0565)
	*BSELF*	0.5608 (0.0729)	0.9357 (1.2401)	0.2828 (0.0463)	2.0844 (0.0277)

**Table 4:** Estimates (Risk of estimates) under *LINEX*, *GELF*, *BLINEX* and *BGELF* with fixed values of, $\varrho _1$ = 0.8, $\varrho _2$ = 5.5, $\rho$ = 2, $\beta$ = 0.1, $\zeta$ = 0.3, $\phi$ = 0.5, *p* = 0.4, $\varepsilon =0.5$.

(n,m)	Method	$k_{1}=k_{2}=$-0.5				$k_{1}=k_{2}=$0.5			
		${\hat{\zeta }}$	${\hat{\phi }}$	${\hat{\beta }}$	${\hat{\rho }}$	${\hat{\zeta }}$	${\hat{\phi }}$	${\hat{\beta }}$	${\hat{\rho }}$
(100,40)	*LINEX*	0.5448 (0.067)	0.7876 (0.1117)	0.2197 (0.0234)	2.1282 (0.0592)	0.5423 (0.0657)	0.7765 (0.1038)	0.2179 (0.0226)	2.1198 (0.0559)
	*GELF*	0.5413 (0.0652)	0.775 (0.1033)	0.2155 (0.0219)	2.1221 (0.0568)	0.5367 (0.0629)	0.7607 (0.0947)	0.209 (0.0196)	2.1182 (0.0555)
	*BLINEX*	0.5078 (0.0483)	0.8202 (0.6505)	0.1942 (0.0149)	2.0932 (0.0315)	0.504 (0.0465)	0.6435 (0.0965)	0.1917 (0.0139)	2.081 (0.0262)
	*BGELF*	0.5023 (0.0457)	0.5699 (0.1429)	0.188 (0.0129)	2.0842 (0.0275)	0.4953 (0.0426)	0.2585 (0.2319)	0.1785 (0.0103)	2.0787 (0.0257)
(100,30)	*LINEX*	0.6124 (0.1045)	0.8095 (0.1256)	0.3395 (0.0775)	2.1219 (0.0572)	0.6092 (0.1025)	0.7969 (0.1161)	0.3339 (0.0734)	2.1134 (0.054)
	*GELF*	0.6082 (0.1018)	0.7955 (0.1156)	0.3295 (0.071)	2.1156 (0.055)	0.6027 (0.0984)	0.7797 (0.1054)	0.3143 (0.0622)	2.1117 (0.0537)
	*BLINEX*	0.5644 (0.075)	1.4067 (8.9641)	0.2888 (0.0495)	2.0882 (0.0293)	0.5588 (0.0718)	0.6949 (0.1598)	0.2805 (0.0448)	2.0764 (0.0254)
	*BGELF*	0.5567 (0.0707)	0.6949 (0.5144)	0.2722 (0.0412)	2.0795 (0.0264)	0.5468 (0.0655)	0.2679 (0.2809)	0.2477 (0.0311)	2.0741 (0.0249)
(100,20)	*LINEX*	0.6418 (0.1214)	0.8146 (0.1286)	0.4828 (0.1708)	2.1252 (0.0597)	0.6386 (0.1191)	0.8018 (0.1188)	0.4702 (0.159)	2.1166 (0.0564)
	*GELF*	0.6376 (0.1185)	0.8003 (0.1183)	0.4635 (0.154)	2.1189 (0.0574)	0.6322 (0.1148)	0.7842 (0.1079)	0.4359 (0.1324)	2.1149 (0.0561)
	*BLINEX*	0.6032 (0.0952)	1.3961 (12.0121)	0.4344 (0.1846)	2.0907 (0.0306)	0.5979 (0.0919)	0.736 (0.2214)	0.4116 (0.121)	2.0785 (0.0264)
	*BGELF*	0.5961 (0.0909)	1.1919 (5.0528)	0.4007 (0.1164)	2.0817 (0.0276)	0.5871 (0.0856)	0.2992 (0.3455)	0.3589 (0.0884)	2.0761 (0.026)
(200,80)	*LINEX*	0.6124 (0.1045)	0.8095 (0.1256)	0.3395 (0.0775)	2.1219 (0.0572)	0.6092 (0.1025)	0.7969 (0.1161)	0.3339 (0.0734)	2.1134 (0.054)
	*GELF*	0.6082 (0.1018)	0.7955 (0.1156)	0.3295 (0.071)	2.1156 (0.055)	0.6027 (0.0984)	0.7797 (0.1054)	0.3143 (0.0622)	2.1117 (0.0537)
	*BLINEX*	0.5644 (0.075)	1.4067 (8.9641)	0.2888 (0.0495)	2.0882 (0.0293)	0.5588 (0.0718)	0.6949 (0.1598)	0.2805 (0.0448)	2.0764 (0.0254)
	*BGELF*	0.5567 (0.0707)	0.6949 (0.5144)	0.2722 (0.0412)	2.0795 (0.0264)	0.5468 (0.0655)	0.2679 (0.2809)	0.2477 (0.0311)	2.0741 (0.0249)
(200,50)	*LINEX*	0.4862 (0.0397)	0.7682 (0.0987)	0.1589 (0.0065)	2.1199 (0.0561)	0.4844 (0.039)	0.7583 (0.0921)	0.1583 (0.0064)	2.1121 (0.0533)
	*GELF*	0.4835 (0.0386)	0.7569 (0.0916)	0.1571 (0.0062)	2.1142 (0.0541)	0.4798 (0.0372)	0.7439 (0.0843)	0.1542 (0.0057)	2.1106 (0.053)
	*BLINEX*	0.4618 (0.0299)	0.687 (0.1364)	0.1481 (0.0045)	2.0868 (0.0288)	0.4596 (0.029)	0.6099 (0.0607)	0.1474 (0.0043)	2.0756 (0.025)
	*BGELF*	0.4584 (0.0286)	0.5321 (0.0742)	0.146 (0.0041)	2.0786 (0.026)	0.4538 (0.027)	0.2761 (0.1958)	0.1426 (0.0035)	2.0734 (0.0245)
(200,30)	*LINEX*	0.6117 (0.104)	0.8082 (0.1244)	0.3373 (0.0761)	2.1248 (0.0582)	0.6086 (0.1019)	0.7958 (0.1151)	0.3318 (0.0722)	2.1163 (0.0549)
	*GELF*	0.6075 (0.1013)	0.7944 (0.1146)	0.3274 (0.0698)	2.1186 (0.0559)	0.6022 (0.098)	0.7787 (0.1046)	0.3124 (0.0612)	2.1146 (0.0546)
	*BLINEX*	0.5636 (0.0745)	1.3726 (7.9233)	0.2869 (0.0487)	2.0903 (0.0298)	0.558 (0.0713)	0.6943 (0.1586)	0.2787 (0.0441)	2.0784 (0.0257)
	*BGELF*	0.5559 (0.0702)	0.6899 (0.4862)	0.2705 (0.0405)	2.0816 (0.0269)	0.546 (0.0649)	0.2691 (0.2796)	0.2462 (0.0305)	2.0761 (0.0252)

**Table 5:** AL (CP) of estimates under *MLE*, *MAP* and BAYS approach.

		$\varrho _1$ = 0.8, $\varrho _2$5.5, $\rho$ = 2, $\beta$ = 0.1, $\zeta$ = 0.3, $\phi$ = 0.5, *p* = 0.4				$\varrho _1$ = 1.5, $\varrho _2$6, $\rho$ = 2, $\beta$ = 0.1, $\zeta$ = 0.3, $\phi$ = 0.5, *p* = 0.4			
(n,m)	Method	${\hat{\zeta }}$	${\hat{\phi }}$	${\hat{\beta }}$	${\hat{\rho }}$	${\hat{\zeta }}$	${\hat{\phi }}$	${\hat{\beta }}$	$\rho$
(100,40)	*MLE*	0.8757 (0.4534)	1.8177 (0.9919)	0.1509 (0.9753)	2.4619 (0.591)	0.673 (J50.8926)	1.5597 (0.964)	0.1823 (0.9409)	3.0178 (0.7236)
	*MAP*	0.6706 (0.9277)	2.4037 (0.9447)	0.1696 (0.9617)	11.1362 (0.8249)	0.6409 (0.4254)	1.0764 (1)	0.1498 (0.9477)	3.5314 (0.8867)
	BAYS	0.2482 (0.9326)	0.4707 (0.9312)	0.1867 (0.9411)	0.4126 (0.9114)	0.2572 (0.9668)	0.5189 (0.9717)	0.2187 (0.9809)	0.3849 (0.9745)
(100,30)	*MLE*	0.9096 (0.4749)	1.8763 (0.9904)	0.1534 (0.9836)	2.6258 (0.6323)	0.7127 (0.9037)	1.8281 (0.976)	0.2056 (0.9491)	3.3185 (0.7954)
	*MAP*	0.705 (0.9324)	2.834 (0.9553)	1.6611 (0.9617)	34.7466 (0.8306)	0.6944 (0.4239)	1.0999 (0.9999)	0.1402 (0.9834)	4.0799 (0.9394)
	BAYS	0.2765 (0.9332)	0.4965 (0.9977)	0.3248 (0.9556)	0.4163 (0.9805)	0.2764 (0.9477)	0.4951 (0.9829)	0.3233 (0.9879)	0.4154 (0.97)
(100,20)	*MLE*	1.0909 (0.4237)	2.0667 (0.9779)	0.1534 (0.9957)	3.2227 (0.7593)	0.8402 (0.9081)	2.2804 (0.9786)	26.282 (0.9881)	4.7547 (0.9276)
	*MAP*	0.7837 (0.9367)	3.7714 (0.9633)	1.7326 (0.1927)	53 (0.9124)	1.2427 (0.2261)	1.2833 (1)	0.0986 (0.8324)	4.8621 (0.9693)
	BAYS	0.283 (0.9189)	0.5034 (0.967)	0.4935 (0.9703)	0.4191 (0.9351)	0.2829 (0.9172)	0.5015 (0.9758)	0.4899 (0.9521)	0.4176 (0.9667)
(200,80)	*MLE*	0.4214 (0.6487)	1.3817 (0.999)	0.104 (0.964)	2.9547 (0.7393)	0.4387 (0.8831)	0.9479 (0.971)	0.1282 (0.9361)	2.1782 (0.6119)
	*MAP*	0.4182 (0.9067)	1.446 (0.9446)	0.1145 (0.961)	3.8914 (0.753)	0.3861 (0.5467)	0.7742 (0.9986)	0.1147 (0.9226)	2.8868 (0.8529)
	BAYS	0.2765 (0.912)	0.4965 (0.9939)	0.3248 (0.9399)	0.4163 (0.9767)	0.1751 (0.9587)	0.3651 (0.9797)	0.0543 (0.9719)	0.3712 (0.9236)
(200,50)	*MLE*	0.4313 (0.709)	1.4438 (0.9981)	0.1097 (0.9833)	2.9923 (0.7669)	0.4656 (0.8963)	1.1526 (0.9821)	0.1427 (0.9489)	2.6682 (0.7586)
	*MAP*	0.4522 (0.9087)	1.9917 (0.9687)	0.14 (0.9499)	4.3561 (0.7699)	0.3999 (0.6001)	0.7983 (0.9991)	0.1095 (0.9883)	4.058 (0.9771)
	BAYS	0.218 (0.9375)	0.4444 (0.9477)	0.114 (0.984)	0.3973 (0.9982)	0.2185 (0.9497)	0.4469 (0.9336)	0.1132 (0.9781)	0.3972 (0.9457)
(200,30)	*MLE*	0.5103 (0.6774)	1.667 (0.9971)	0.0976 (0.9836)	4.1989 (0.9403)	0.5775 (0.9017)	1.582 (0.9861)	0.1289 (0.957)	42.1205 (0.9389)
	*MAP*	0.5136 (0.928)	3.0411 (0.9737)	0.1978 (0.9901)	5.13338 (0.8727)	0.4023 (0.6321)	0.8123 (0.9997)	0.1081 (0.9989)	6.1253 (0.9978)
	BAYS	0.2761 (0.9689)	0.496 (0.9577)	0.3217 (0.9424)	0.417 (0.92)	0.276 (0.9647)	0.4958 (0.9603)	0.3263 (0.9915)	0.4167 (0.9977)

### Application of the failure times of aircraft windshields data

**Fig. 2:**
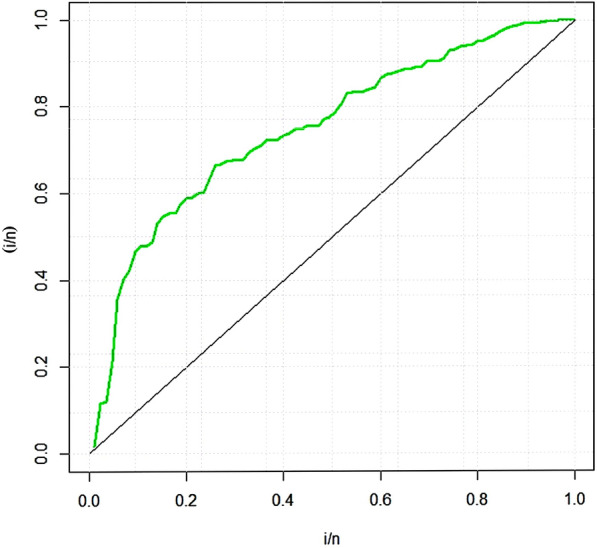
Estimated TTT-transform plot for the Aircraft windshields data.

To illustrate the value of the suggested distribution for *SSPALT* under $PT\text {-}II CBRs$, we examine a real dataset in this section. The dataset was recently studied by^36^. The data set represents the failure times of $n =$ 85 observations on the Aircraft windshields. The data set along with summary statistics is provided by Table 6. The TTT (Transformation-Time-Transformed) plot is used to analyze failure rates by transforming the data^37^. The TTT transformation curves of the data depicted by Fig. 2 suggest an increasing failure rate, implying that the likelihood of failure rises over time. Therefore, the *MWD*, due to its flexibility in handling varying failure rates, is the most suitable model for fitting this dataset.

The dataset is fitted using the *MLE* approach and it is then contrasted with different competing distributions, specifically:

- Weibull distribution (WD)
- Exponential distribution (ED)
- Exponentiated Weibull (Ex-weibuII) distribution by^38^
- Inverse Weibull Weibull (IW-weibuII) distribution by^39^
- Exponinated Kumaraswamy–Weibull (ExKu-weibuII) distribution by^40^
- Gamma distribution (GD)
- GIKw-Weibull distribution by^41^
- Burr-XII distribution
- Kumaraswamy Exponential-Weibull (KuE-weibuII) distribution by^42^
- GIKw-Weibull distribution by^43^
- Generalized Inverse Weibull distribution (GIWD) by^44^
- *MWD*

**Fig. 3:**
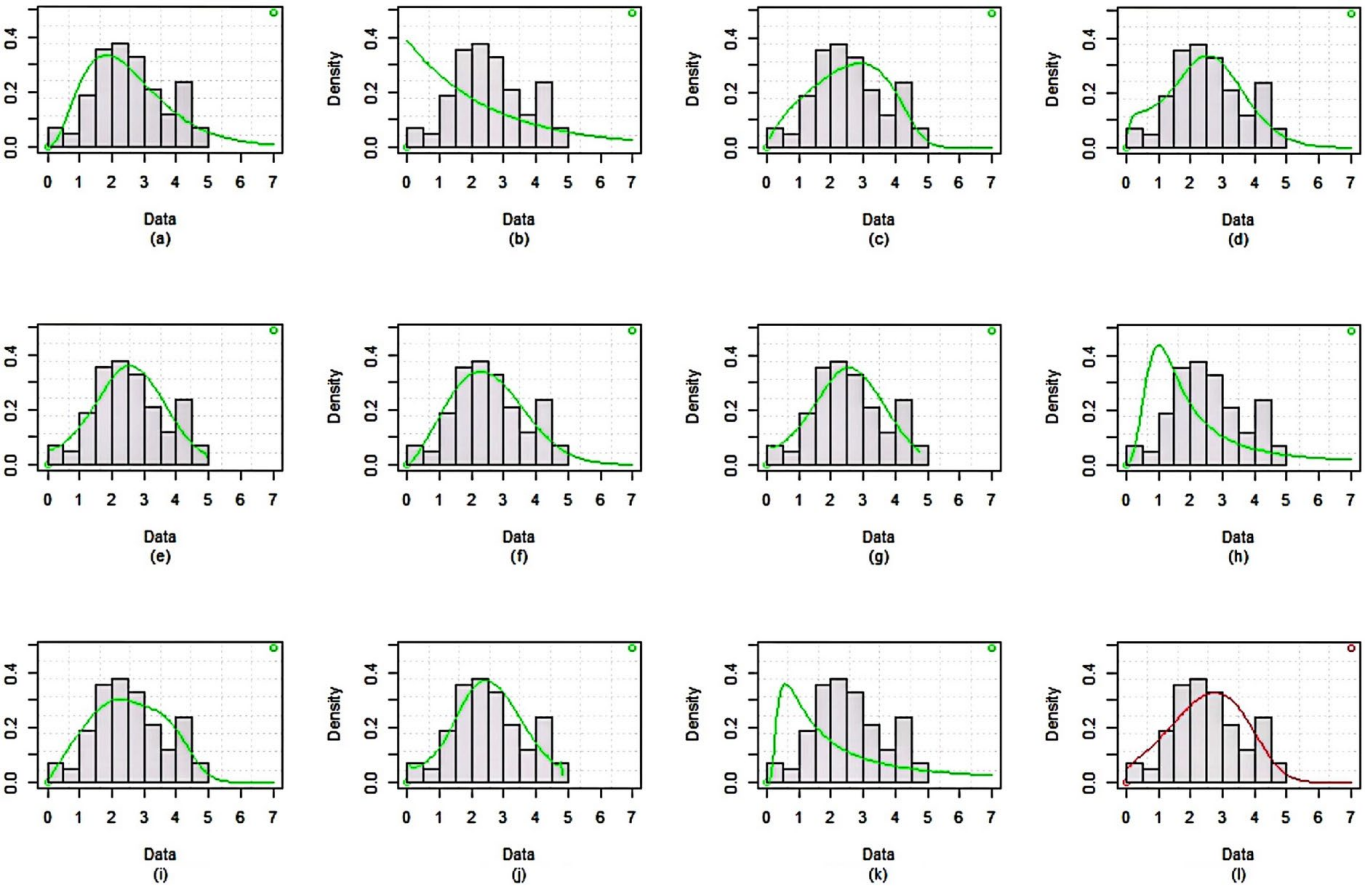
Estimated histograms of the considered models for the Aircraft windshields data.

**Fig. 4:**
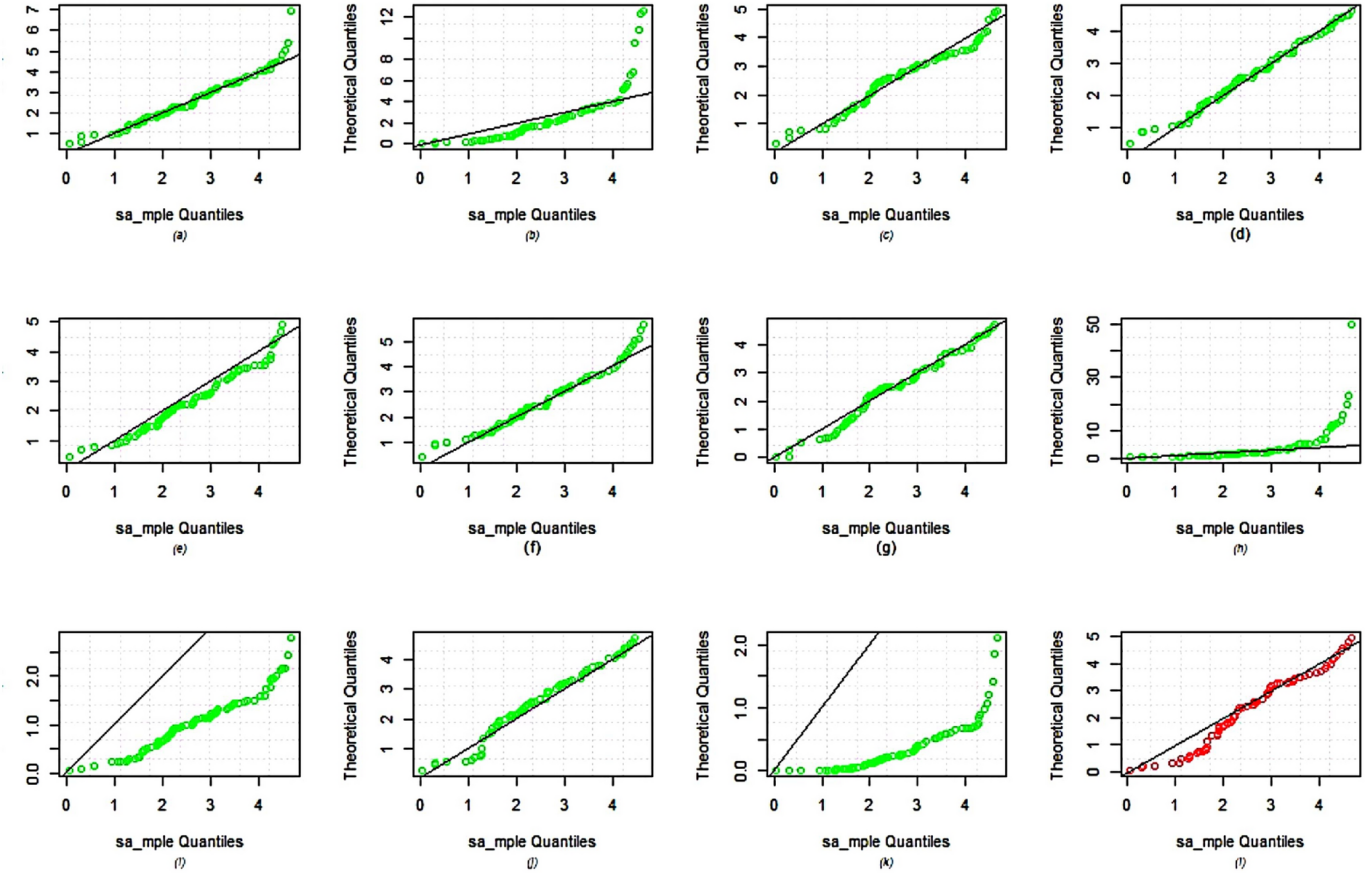
Normal QQ plots of the considered models for the Aircraft windshields data.

**Fig. 5:**
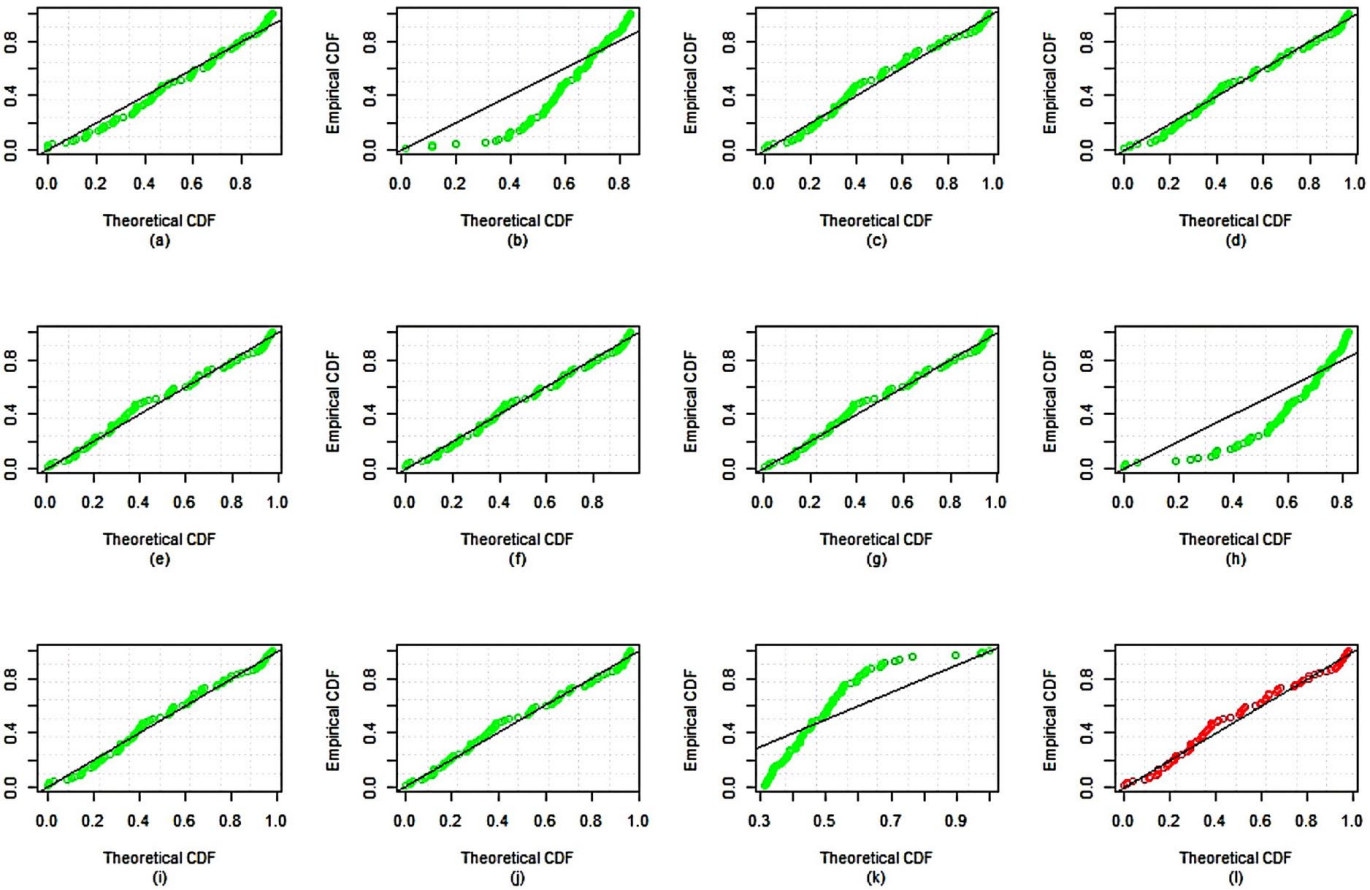
Normal *PP* plots of the considered models for the Aircraft windshields data.

**Fig. 6:**
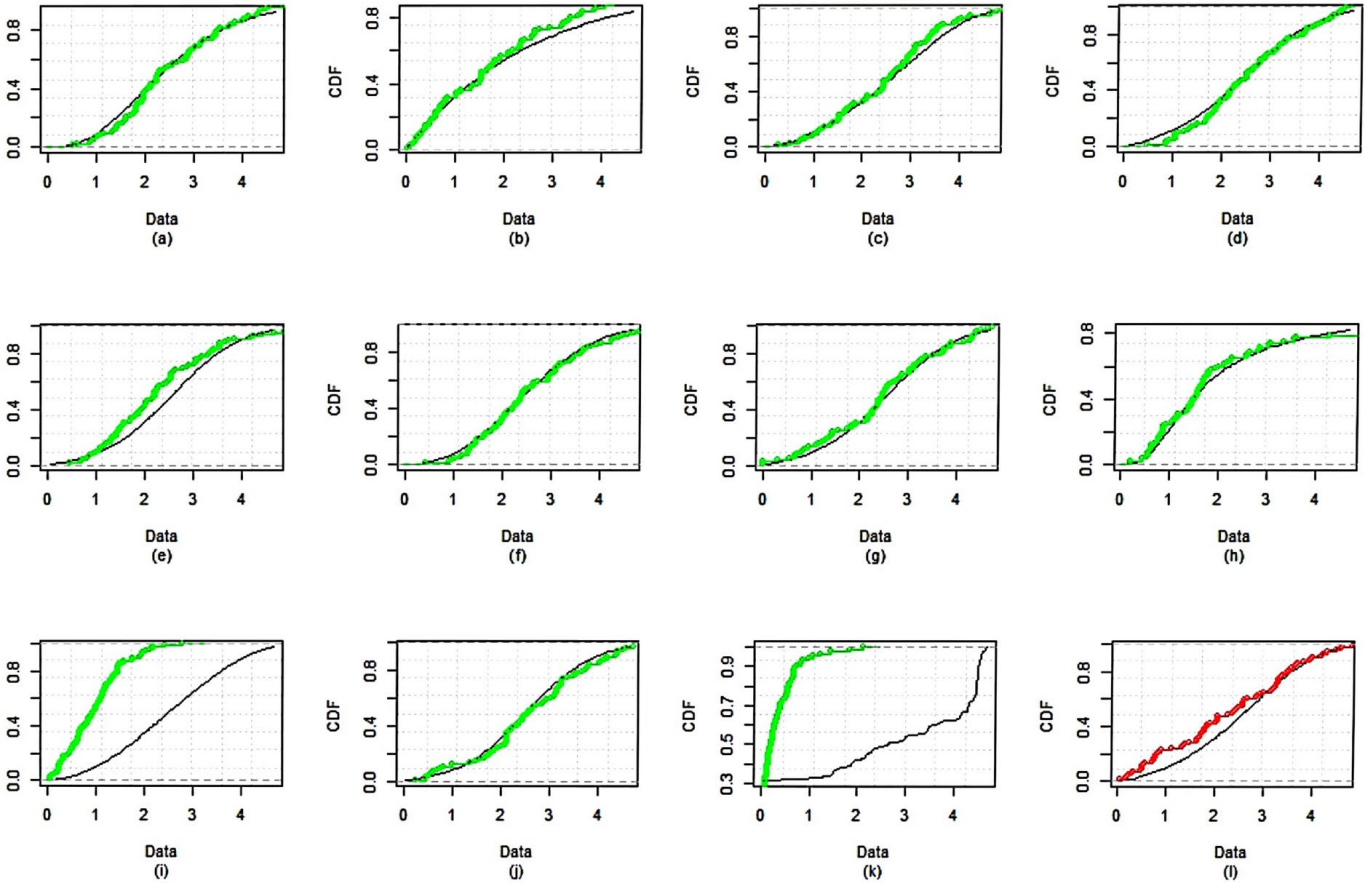
Estimated *CDF* plots of the considered models for the Aircraft windshields data.

The objective of this comparative analysis is to evaluate the goodness-of-fit of various competing distributions to the given dataset, with a particular focus on justifying the selection of the *MWD* over the conventional Weibull distribution and other considered models. To assess the adequacy of these distributions, we employed multiple graphical diagnostics, including sample histogram plots (Fig. 3), quantile-quantile ($Q-Q$) plots (Fig. 4), probability-probability ($P-P$) plots (Fig. 5), and estimated cumulative distribution function (*CDF*) plots (Fig. 6). The visual assessments strongly indicate that the *MWD* provides a superior fit to the observed data compared to the standard Weibull and other higher-parametric distributions. This suggests that the additional flexibility of the *MWD* allows it to capture the data characteristics more effectively than the conventional Weibull model. Further statistical validation is provided in Table 19, which presents the maximum likelihood estimates (*MLE*) of model parameters for all distributions, along with key goodness-of-fit criteria, including the Kolmogorov–Smirnov (*K*-*S*) statistic with its corresponding *P*-value, the Akaike Information Criterion (*AIC*), the Corrected Akaike Information Criterion (*CAIC*), the Bayesian Information Criterion (*BIC*), and the Hannan–Quinn Information Criterion (*HQIC*). The results reveal that the *MWD* exhibits the lowest values for *AIC*, *CAIC*, *BIC*, and *HQIC*, demonstrating its superior model fit. Additionally, the *K*-*S* statistic for the *MWD* is among the smallest recorded values, reinforcing its robustness in capturing the underlying data distribution. Notably, the Weibull distribution, despite being a widely used reliability model, fails to achieve the same level of goodness-of-fit as the *MWD*. The substantial reduction in information criteria values when using the *MWD* highlights its enhanced flexibility and better adaptability to real-world failure time data. These findings unequivocally establish the *MWD* as the most suitable model among all the considered distributions, providing strong empirical evidence for its application in reliability analysis. The *SSPALT* under $PT\text {-}II CBRs$ is constructed using five schemes for each of the three considered cases of $\varrho _1$, $\varrho _2$ and *p* for the given *n*=84 observations of Aircraft windshields data. The values $\varrho _1 = 1.4, 1.8, 1.5$, $\varrho _2 = 4.2, 4.6, 4.5$, $\rho = 2$, $\beta = 0.1$, $\zeta = 0.3$, $\phi = 0.5$, and $p = 0.4$ were chosen based on the empirical characteristics of the dataset (Table 6) and the theoretical framework of the MWD in SSALT context. Specifically, $\varrho _1$, the stress change parameter, was set between 1.0 and 2.0, and $\varrho _2$, the experiment execution time parameter, between 4.0 and 5.0, to ensure a reasonable number of failures occur before the stress shift at $\varrho _1$ and experiment completion at $\varrho _2$. This decision was informed by summary measures in Table 6 (e.g., mean = 2.5626, max = 4.663, skewness = 0.0074), ensuring alignment with the dataset?s failure time distribution and practical testing constraints. The core parameters $\rho = 2$, $\beta = 0.1$, $\zeta = 0.3$, and $\phi = 0.5$ were strategically fixed through preliminary exploratory analysis, reflecting the increasing failure rate observed in the TTT plot (Fig. 2) and consistent with the physical context of windshield failures. Here, $\rho = 2$ serves as the acceleration factor in the TRV model (Eq. ), a critical component of SSALT that amplifies stress levels, while $\zeta = 0.3$ modulates the hazard function?s shape, and $\beta = 0.1$ and $\phi = 0.5$ adjust the scale, all validated against the empirical distribution via MLE, MAP, and BAYS estimates. The removal probability $p = 0.4$ in the binomial framework for progressive type-II censoring was set to achieve a reasonable number of unit removals during the experiment. This constant probability, applied across all stress levels, balances the need to retain enough units for analysis while simulating realistic censoring, consistent with reliability testing practices. This rigorous approach ensures that the fixed values are not only data-driven but also theoretically sound, providing a robust foundation for the estimation results presented. Any apparent deviations in the intervals from these fixed values are addressed in the revised BAYS results, as discussed previously, reinforcing the methodological integrity of our analysis.It demonstrates the methodology covered in the aforementioned sections of this article.

**Fig. 7:**
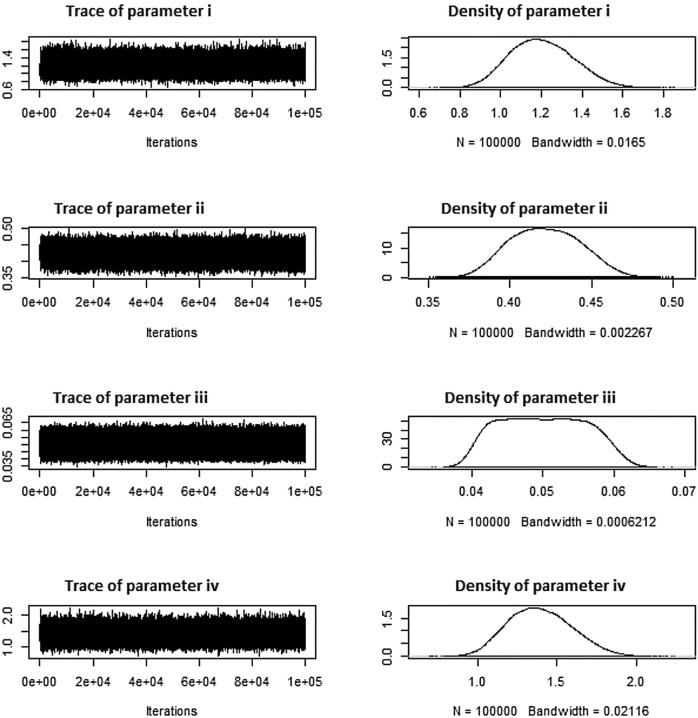
The *MCMC* trace and density plots of $\zeta , \phi$ and $\beta$ for the Aircraft windshields data at $\varrho _1 = 1.5, \varrho _2 = 4.5, \rho = 2, \beta = 0.1, \zeta = 0.3, \phi = 0.5, p = 0.4$.

**Fig. 8:**
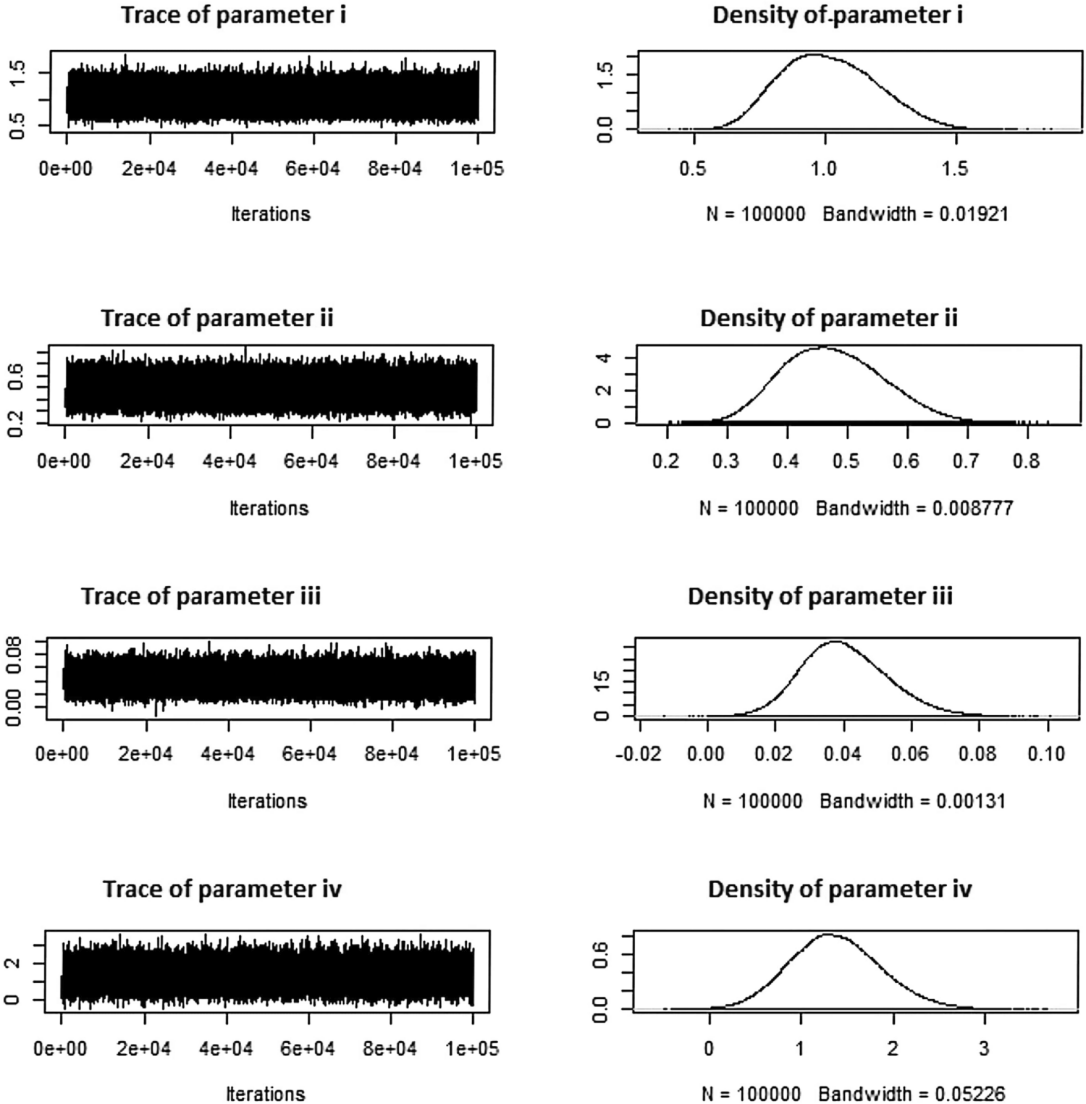
The *MCMC* trace and density plots of $\zeta$, $\phi$ and $\beta$ for the Aircraft windshields data at $\varrho _1$ = 1.4, $\varrho _2$ = 4.2, $\rho$ = 2, $\beta$ = 0.1, $\zeta$ = 0.3, $\phi$ = 0.5, *p* = 0.4.

**Fig. 9:**
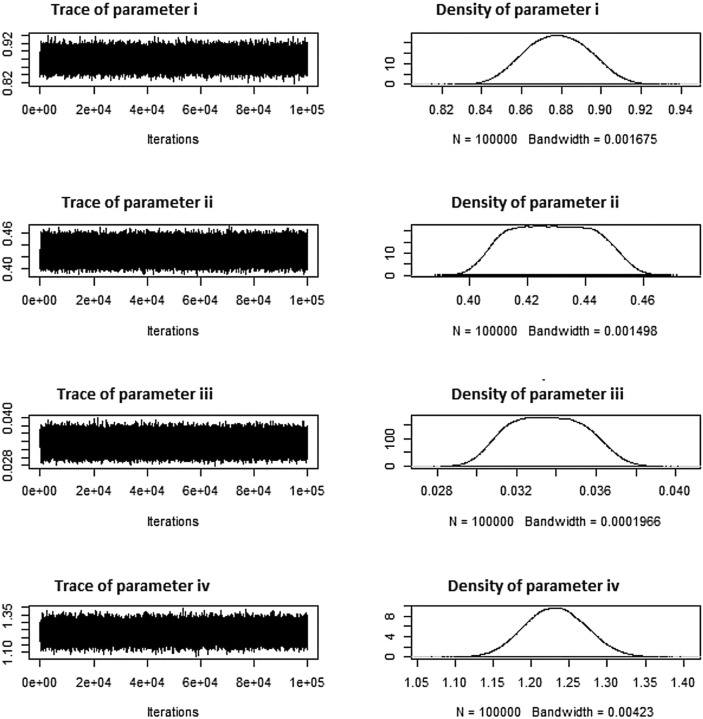
The *MCMC* trace and density plots of $\zeta$, $\phi$ and $\beta$ for the Aircraft windshields data at $\varrho _1$ = 1.8, $\varrho _2$ = 4.6, $\rho$ = 2, $\beta$ = 0.1, $\zeta$ = 0.3, $\phi$ = 0.5, *p* = 0.4.

Tables 16, 17, 18, respectively, display these systems. Tables 16, 17, 18 additionally indicate the number of removals for each dataset under various approaches. The simulation numbers, against diverse parametric combinations, of estimated parameters $\zeta =i$, $\phi =ii$ and $\beta =iii$ and $\rho =iv$ produced using *MCMC* samples are shown in Figs. 7, 8, 9, together with matching density plots for the Aircraft windshields data. Trace plots show well mixing draws and symmetric bell shaped behavior. Estimates under *MLE*, *MAP*, *SELF* and *BSELF* for three considered combinations of $\varrho _1$, $\varrho _2$, $\rho$, $\beta$, $\zeta$, $\phi$ and *p* are presented in Tables 7, 8, 9, respectively. However, in all considered situations the loss parameters for asymmetric loss functions were set at ($k_{1}=k_{2}$= 0.5,-0.5) and the weight parameter for balanced loss functions was used as $\varepsilon =0.5$. Likewise, Tables 10, 11, 12, respectively offer the estimates under *LINEX*, *GELF*, *BLINEX* and *BGELF* for all the three considered parametric combinations of $\varrho _1$, $\varrho _2$, $\rho$, $\beta$, $\zeta$, $\phi$ and *p*. Tables 13, 14, 15, offer the *CI* and *HPD* intervals of estimates under *MLE*, *MAP* and BAYS approach for the various values of model parameters for the Aircraft windshields data. All of these findings are obtained using the formulas given in sections “Likelihood estimation of model parameters” and “Bayesian estimation” under various degrees of censoring. Tables 7, 8, 9, 10, 11, 12, 13, 14, 15 show that as the degree of censoring lowers, the *MLE* and Bayes estimators, together with the length of *CI*/*HPD* intervals of estimators, decrease, respectively. We can witness from the behaviour of the *CI*/*HPD* for all considered scanerios, the average interval length decreases as *m* grows.

Notably, among all estimation approachs, the Bayesian *HPD* in Tables 7, 8, 9, 10, 11, 12, 13, 14, 15 are narrower than those of MLE and MAP, as expected, due to the incorporation of informative priors that enhance precision in parameter estimation. For parameters $\rho$, $\beta$, $\zeta$, $\phi$, the BAYS intervals align closely with or encompass the true values ($\rho =2$, $\beta =0.1$, $\zeta =0.3$, $\phi =0.5$), reflecting the robustness of the prior specification tailored to the Aircraft windshields data. Minor deviations from the true values, such as slight shifts in ${\hat{\rho }}$ for large $m=3$ (e.g., ${\hat{\rho }}$ for $m=30$ in Table 7) can be attributed to the influence of the specific sample characteristics and censoring effects, which may shift posterior distributions. These results affirm the BAYS approach?s superior precision and reliability over MLE and MAP for this dataset.

**Table 6:** The Aircraft windshields dataset alongwith summary measures.

n = 85, Mean = 2.5626, Min = 0.04, Max = 4.663, Var = 1.2391, S.D = 1.1131, C.V = 0.4343, Skew = 0.0074, Kur = 2.3654										
4.663	3.103	2.224	3.117	4.485	3.478	1.248	4.24	1.506	2.61	3.779
0.04	1.866	2.385	3.443	0.301	1.876	2.481	3.467	0.309	1.899	2.934
1.912	2.632	3.595	1.07	1.914	2.646	3.699	1.124	1.981	2.661	4.278
1.281	2.085	2.89	4.121	1.303	2.089	2.902	4.167	1.432	2.097	4.035
2.154	4.376	1.615	2.223	3.114	4.449	1.619	2.82	3	2.964	3.376
0.557	1.911	2.625	3.578	0.943	2.01	2.688	3.924	1.281	2.038	2.324
1.48	2.135	2.962	4.255	1.505	2.19	3	4.305	1.568	2.194	1.757
1.652	2.229	3.166	4.57	1.652	2.3	3.344	4.602

**Table 7:** Estimates under *MLE*, *MAP*, *SELF* and *BSELF* with fixed value of, $\varrho _1$ = 1.5, $\varrho _2$ = 4.5, $\rho$ = 2, $\beta$ = 0.1, $\zeta$ = 0.3, $\phi$ = 0.5, *p* = 0.4, $\varepsilon =0.5$ for the Aircraft windshields data.

m	Method	${\hat{\zeta }}$	${\hat{\phi }}$	${\hat{\beta }}$	${\hat{\rho }}$
20	*MLE*	0.6232	0.8705	0.0316	1.447
	*MAP*	0.6669	0.7685	0.0345	2.7877
	*SELF*	1.3217	0.4665	0.0552	1.1375
	*BSELF*	1.1156	0.5767	0.0486	1.2419
22	*MLE*	0.5739	1.0663	0.0266	1.273
	*MAP*	0.6296	0.8628	0.0315	1.3494
	*SELF*	1.2027	0.2443	0.0749	2.1374
	*BSELF*	1.0005	0.5152	0.0593	1.8993
27	*MLE*	0.4947	1.2757	0.017	1.1997
	*MAP*	0.5323	1.0771	0.0219	1.6005
	*SELF*	0.7172	0.6224	0.046	1.9696
	*BSELF*	0.6781	0.7514	0.0403	1.7459
30	*MLE*	0.5919	0.8896	0.0303	1.5941
	*MAP*	0.675	0.7732	0.0349	1.7071
	*SELF*	0.8063	0.4756	0.066	2.0935
	*BSELF*	0.7608	0.574	0.0568	1.972
40	*MLE*	1.3304	0.0336	0.0907	2.3017
	*MAP*	1.0029	0.185	0.0699	3.2533
	*SELF*	1.2783	0.45	0.0575	1.3155
	*BSELF*	1.2144	0.383	0.0613	1.6258

**Table 8:** Estimates under *MLE*, *MAP*, *SELF* and *BSELF* with fixed value of, $\varrho _1$ = 1.4, $\varrho _2$ = 4.2, $\rho$ = 2, $\beta$ = 0.1, $\zeta$ = 0.3, $\phi$ = 0.5, *p* = 0.4, $\varepsilon =0.5$ for the Aircraft windshields data.

*m*	Method	${\hat{\zeta }}$	${\hat{\phi }}$	${\hat{\beta }}$	${\hat{\rho }}$
20	*MLE*	0.6818	0.7742	0.037	1.3502
	*MAP*	0.6296	0.8628	0.0315	1.3494
	*SELF*	0.5832	0.6387	0.0471	1.865
	*BSELF*	0.629	0.6089	0.0462	1.9136
22	*MLE*	0.7429	0.5231	0.0453	2.0054
	*MAP*	0.5323	1.0771	0.0219	1.6005
	*SELF*	0.8193	0.2364	0.0784	2.7273
	*BSELF*	0.7708	0.4116	0.0652	2.3148
27	*MLE*	0.497	1.2475	0.0178	1.3108
	*MAP*	0.675	0.7732	0.0349	1.7071
	*SELF*	0.8012	0.8308	0.0304	1.8236
	*BSELF*	0.7466	0.8436	0.0306	1.7624
30	*MLE*	1.0343	0.1039	0.0747	2.7586
	*MAP*	1.0029	0.185	0.0699	3.2533
	*SELF*	0.4817	0.8079	0.0409	1.6565
	*BSELF*	0.5073	0.8568	0.0368	1.5765
40	*MLE*	1.0192	0.1153	0.0727	3.5991
	*MAP*	0.6195	0.9257	0.0282	1.4493
	*SELF*	0.4908	1.3093	0.0242	0.827
	*BSELF*	0.4336	1.0665	0.0469	1.1789

**Table 9:** Estimates under *MLE*, *MAP*, *SELF* and *BSELF* with fixed value of, $\varrho _1$ = 1.8, $\varrho _2$ = 4.6, $\rho$ = 2, $\beta$ = 0.1, $\zeta$ = 0.3, $\phi$ = 0.5, *p* = 0.4, $\varepsilon =0.5$ for the Aircraft windshields data.

*m*	Method	${\hat{\zeta }}$	${\hat{\phi }}$	${\hat{\beta }}$	${\hat{\rho }}$
20	*MLE*	0.5776	1.0668	0.0268	1.4643
	*MAP*	0.526	1.32	0.0204	0.6525
	*SELF*	0.9002	0.69	0.0458	2.1619
	*BSELF*	0.7962	0.82	0.0394	2.0015
22	*MLE*	0.5393	1.1075	0.0238	2.2555
	*MAP*	0.5198	1.23	0.018	2.8403
	*SELF*	0.5486	1.1	0.0292	1.5595
	*BSELF*	0.5492	1.11	0.0277	1.6011
27	*MLE*	0.5637	1.1497	0.025	1.1974
	*MAP*	0.703	0.74	0.0368	1.9546
	*SELF*	0.7646	0.89	0.0356	1.8519
	*BSELF*	0.7005	0.95	0.0324	1.7783
30	*MLE*	0.5771	1.1338	0.0259	1.308
	*MAP*	0.5602	1.09	0.0241	1.6206
	*SELF*	0.7441	0.89	0.0406	1.2245
	*BSELF*	0.6803	0.97	0.0352	1.2332
40	*MLE*	0.6319	1.0566	0.0286	0.8172
	*MAP*	0.7249	0.89	0.035	0.8643
	*SELF*	0.7259	0.99	0.0344	0.7879
	*BSELF*	0.6913	1.01	0.0324	0.7879

**Table 10:** Estimates under *LINEX*, *GELF*, *BLINEX* and *BGELF* with fixed values of, $\varrho _1$ = 1.5, $\varrho _2$ = 4.5, $\rho$ = 2, $\beta$ = 0.1, $\zeta$ = 0.3, $\phi$ = 0.5, *p* = 0.4, $\varepsilon =0.5$ for the Aircraft windshields data.

m	Method	$k_{1}=k_{2}=-0.5$				$k_{1}=k_{2}=0.5$			
		${\hat{\zeta }}$	${\hat{\phi }}$	${\hat{\beta }}$	${\hat{\rho }}$	${\hat{\zeta }}$	${\hat{\phi }}$	${\hat{\beta }}$	${\hat{\rho }}$
20	*GELF*	1.2459	0.3524	0.0479	1.1194	1.3	0.4397	0.0528	1.1315
	*LINEX*	1.2948	0.4562	0.0551	1.1308	1.35	0.4768	0.0554	1.1441
	*BGELF*	0.9927	0.44	0.0426	1.2136	1.08	0.5448	0.0464	1.2327
	*BLINEX*	1.073	0.5622	0.0485	1.2308	1.16	0.5908	0.0487	1.2528
22	*GELF*	1.0926	0.1418	0.0657	2.0594	1.17	0.2223	0.0717	2.1123
	*LINEX*	1.1665	0.2403	0.0746	2.086	1.24	0.2483	0.0751	2.1863
	*BGELF*	0.8537	0.2185	0.045	1.794	0.95	0.4242	0.0542	1.8635
	*BLINEX*	0.9529	0.472	0.059	1.833	1.05	0.5627	0.0596	1.9686
27	*GELF*	0.6639	0.6105	0.0424	1.9563	0.7	0.6185	0.0448	1.965
	*LINEX*	0.7068	0.6201	0.046	1.9603	0.73	0.6247	0.0461	1.9803
	*BGELF*	0.6393	0.7082	0.0366	1.6794	0.67	0.7367	0.039	1.7243
	*BLINEX*	0.6702	0.7403	0.0403	1.7095	0.69	0.7629	0.0404	1.7818
30	*GELF*	0.7898	0.4214	0.0547	2.0849	0.8	0.4577	0.0624	2.0907
	*LINEX*	0.8019	0.4672	0.0658	2.0877	0.81	0.4843	0.0663	2.0993
	*BGELF*	0.7451	0.5009	0.0475	1.9525	0.76	0.5513	0.0535	1.9655
	*BLINEX*	0.7565	0.5623	0.0566	1.9593	0.77	0.5855	0.057	1.9847
40	*GELF*	1.1143	0.3278	0.0517	1.2992	1.23	0.4112	0.0555	1.3101
	*LINEX*	1.2207	0.4337	0.0573	1.3085	1.33	0.4671	0.0576	1.3225
	*BGELF*	1.0992	0.2913	0.0564	1.5248	1.18	0.3501	0.0598	1.5904
	*BLINEX*	1.1727	0.3693	0.0612	1.5682	1.26	0.3979	0.0614	1.6895

**Table 11:** Estimates under *LINEX*, *GELF*, *BLINEX* and *BGELF* with fixed values of, $\varrho _1$ = 1.4, $\varrho _2$ = 4.2, $\rho$ = 2, $\beta$ = 0.1, $\zeta$ = 0.3, $\phi$ = 0.5, *p* = 0.4, $\varepsilon =0.5$ for the Aircraft windshields data.

m	Method	$k_{1}=k_{2}=-0.5$				$k_{1}=k_{2}=0.5$			
		${\hat{\zeta }}$	${\hat{\phi }}$	${\hat{\beta }}$	${\hat{\rho }}$	${\hat{\zeta }}$	${\hat{\phi }}$	${\hat{\beta }}$	${\hat{\rho }}$
20	*GELF*	0.5308	0.5692	0.0403	1.8539	0.57	0.6172	0.0448	1.8613
	*LINEX*	0.5746	0.6263	0.047	1.8582	0.59	0.6509	0.0472	1.8719
	*BGELF*	0.5823	0.5601	0.0414	1.9034	0.62	0.5934	0.0445	1.9102
	*BLINEX*	0.6217	0.5999	0.0461	1.9073	0.64	0.6181	0.0463	1.9196
22	*GELF*	0.7916	0.1207	0.068	2.6999	0.81	0.2057	0.0751	2.7182
	*LINEX*	0.8119	0.2299	0.0781	2.7027	0.83	0.2433	0.0786	2.7521
	*BGELF*	0.7474	0.1817	0.054	2.1374	0.76	0.3471	0.0612	2.2588
	*BLINEX*	0.7644	0.3894	0.0649	2.1944	0.78	0.4345	0.0655	2.4247
27	*GELF*	0.7884	0.7917	0.0252	1.799	0.8	0.818	0.0286	1.8154
	*LINEX*	0.7979	0.8205	0.0303	1.8087	0.8	0.8412	0.0305	1.8386
	*BGELF*	0.731	0.8149	0.0268	1.7422	0.74	0.8344	0.0294	1.7555
	*BLINEX*	0.7426	0.8362	0.0306	1.7501	0.75	0.8509	0.0307	1.7754
30	*GELF*	0.4231	0.7682	0.0361	1.6085	0.47	0.7957	0.0392	1.6413
	*LINEX*	0.4749	0.7989	0.0408	1.6325	0.49	0.8166	0.041	1.6795
	*BGELF*	0.4599	0.8217	0.0331	1.5377	0.49	0.8464	0.0354	1.5636
	*BLINEX*	0.5021	0.8489	0.0368	1.5565	0.51	0.8641	0.0369	1.5969
40	*GELF*	0.4062	1.232	0.0189	0.7922	0.47	1.2833	0.0224	0.8154
	*LINEX*	0.4813	1.276	0.0241	0.8175	0.5	1.3442	0.0242	0.8367
	*BGELF*	0.3691	0.8986	0.0275	1.0028	0.41	1.0103	0.0398	1.1159
	*BLINEX*	0.4251	1.01	0.0466	1.105	0.44	1.1261	0.0473	1.2616

**Table 12:** Estimates under *LINEX*, *GELF*, *BLINEX* and *BGELF* with fixed values of, $\varrho _1$ = 1.8, $\varrho _2$ = 4.6, $\rho$ = 2, $\beta$ = 0.1, $\zeta$ = 0.3, $\phi$ = 0.5, *p* = 0.4, $\varepsilon =0.5$ for the Aircraft windshields data.

m	Method	$k_{1}=k_{2}=$-0.5				$k_{1}=k_{2}=$0.5			
		${\hat{\zeta }}$	${\hat{\phi }}$	${\hat{\beta }}$	${\hat{\rho }}$	${\hat{\zeta }}$	${\hat{\phi }}$	${\hat{\beta }}$	${\hat{\rho }}$
20	*GELF*	0.7921	0.6412	0.042	2.1338	0.87	0.6792	0.0445	2.1525
	*LINEX*	0.8743	0.6855	0.0458	2.1416	0.93	0.7036	0.0459	2.183
	*BGELF*	0.7064	0.7468	0.0352	1.9595	0.77	0.8009	0.0379	1.9872
	*BLINEX*	0.7725	0.8072	0.0394	1.9729	0.82	0.8391	0.0395	2.0318
22	*GELF*	0.4738	1.07	0.0258	1.507	0.53	1.0867	0.0281	1.5424
	*LINEX*	0.5392	1.0854	0.0291	1.5336	0.56	1.107	0.0292	1.585
	*BGELF*	0.495	1.0887	0.0253	1.5608	0.53	1.1008	0.0269	1.5883
	*BLINEX*	0.5425	1.0999	0.0276	1.5816	0.56	1.1152	0.0277	1.6196
27	*GELF*	0.6968	0.836	0.0302	1.833	0.74	0.87	0.0337	1.8458
	*LINEX*	0.7486	0.8725	0.0355	1.841	0.78	0.9001	0.0356	1.8623
	*BGELF*	0.6475	0.9038	0.0285	1.7603	0.68	0.9357	0.031	1.7723
	*BLINEX*	0.6872	0.9375	0.0323	1.7677	0.72	0.9616	0.0324	1.789
30	*GELF*	0.7125	0.8497	0.0361	1.2011	0.73	0.8756	0.039	1.2167
	*LINEX*	0.7367	0.8777	0.0405	1.215	0.75	0.8963	0.0407	1.2342
	*BGELF*	0.6495	0.9318	0.0311	1.2165	0.67	0.9613	0.0337	1.2277
	*BLINEX*	0.6729	0.9628	0.0352	1.2265	0.69	0.9846	0.0353	1.24
40	*GELF*	0.7043	0.9663	0.0313	0.7672	0.72	0.9803	0.0333	0.7812
	*LINEX*	0.7212	0.9805	0.0344	0.7828	0.73	0.9934	0.0345	0.7929
	*BGELF*	0.674	0.9984	0.0302	0.7733	0.69	1.0096	0.0316	0.7832
	*BLINEX*	0.6874	1.0098	0.0324	0.7844	0.7	1.0197	0.0325	0.7914

**Table 13:** CI of estimates under MLE, MAP, and BAYS approaches with fixed values of $\varrho _1 = 1.5$, $\varrho _2 = 4.5$, $\rho = 2$, $\beta = 0.1$, $\zeta = 0.3$, $\phi = 0.5$, $p = 0.4$ for the Aircraft windshields data.

m	Method	${\hat{\zeta }}$	${\hat{\phi }}$	${\hat{\beta }}$	${\hat{\rho }}$
20	*MLE*	(0.1222, 3.1789)	(0.169, 4.4843)	(0.0047, 0.2122)	(0.4594, 4.5575)
	MAP	(0.1101, 3.7206)	(0.1841, 5.3499)	(0.0035, 0.2634)	(0.3709, 4.2763)
	BAYS	(0.2856, 0.3143)	(0.4754, 0.5245)	(0.0923, 0.1076)	(1.9552, 2.0447)
22	*MLE*	(0.1048, 3.1427)	(0.284, 4.0033)	(0.004, 0.1773)	(0.4561, 3.5528)
	MAP	(0.123, 3.6165)	(0.0984, 6.4278)	(0.004, 0.295)	(0.4499, 6.6926)
	BAYS	(0.2874, 0.3125)	(0.4792, 0.5207)	(0.0931, 0.1068)	(1.9601, 2.0398)
27	*MLE*	(0.0675, 3.6279)	(0.3722, 4.3724)	(0.002, 0.1438)	(0.4458, 3.2288)
	MAP	(0.1355, 3.7194)	(0.0605, 8.2263)	(0.0043, 0.3394)	(0.4112, 8.4776)
	BAYS	(0.2891, 0.3108)	(0.4776, 0.5223)	(0.0942, 0.1057)	(1.9654, 2.0345)
30	*MLE*	(0.1231, 2.8465)	(0.2072, 3.8194)	(0.0053, 0.1725)	(0.5615, 4.5254)
	MAP	(0.0868, 2.9195)	(0.3784, 3.7157)	(0.0033, 0.1324)	(0.4788, 3.0765)
	BAYS	(0.2869, 0.3130)	(0.4789, 0.5210)	(0.0937, 0.1062)	(1.9703, 2.0296)
40	*MLE*	(0.7059, 2.5074)	(0.0001, 580.554)	(0.0489, 0.1682)	(0.7319, 7.2381)
	MAP	(0.6119, 2.3661)	(0.0003, 15.4868)	(0.0462, 0.1665)	(0.6116, 12.6965)
	BAYS	(0.2887, 0.3112)	(0.4767, 0.5232)	(0.0948, 0.1051)	(1.9752, 2.0247)

**Table 14:** CI of estimates under MLE, MAP, and BAYS approaches with fixed values of $\varrho _1 = 1.4$, $\varrho _2 = 4.2$, $\rho = 2$, $\beta = 0.1$, $\zeta = 0.3$, $\phi = 0.5$, $p = 0.4$ for the Aircraft windshields data.

m	Method	${\hat{\zeta }}$	${\hat{\phi }}$	${\hat{\beta }}$	${\hat{\rho }}$
20	*MLE*	(0.1296, 3.5859)	(0.0874, 6.8552)	(0.0045, 0.3047)	(0.3568, 5.1089)
	MAP	(0.1187, 3.3387)	(0.1301, 5.7209)	(0.004, 0.2504)	(0.3716, 4.9001)
	BAYS	(0.2801, 0.3198)	(0.4702, 0.5297)	(0.0903, 0.1096)	(1.9504, 2.0495)
22	*MLE*	(0.1677, 3.2904)	(0.0253, 10.8072)	(0.0064, 0.3216)	(0.3935, 10.2202)
	MAP	(0.0856, 3.3094)	(0.2247, 5.162)	(0.0025, 0.1922)	(0.4716, 5.4314)
	BAYS	(0.2854, 0.3147)	(0.4801, 0.5198)	(0.0921, 0.1078)	(1.9603, 2.0396)
27	*MLE*	(0.0696, 3.5485)	(0.3366, 4.6242)	(0.0021, 0.1487)	(0.4619, 3.7195)
	MAP	(0.1268, 3.5928)	(0.0761, 7.8602)	(0.0039, 0.313)	(0.383, 7.6098)
	BAYS	(0.2902, 0.3099)	(0.4753, 0.5246)	(0.0945, 0.1054)	(1.9701, 2.0298)
30	*MLE*	(0.4698, 2.277)	(0.0007, 16.3183)	(0.0342, 0.1632)	(0.5661, 13.4421)
	MAP	(0.4667, 2.1549)	(0.0062, 5.4832)	(0.0323, 0.1509)	(0.4917, 21.5239)
	BAYS	(0.2876, 0.3123)	(0.4824, 0.5175)	(0.0932, 0.1067)	(1.9652, 2.0347)
40	*MLE*	(0.4995, 2.0796)	(0.0081, 1.644)	(0.0379, 0.1398)	(0.9577, 13.5253)
	MAP	(0.097, 3.958)	(0.1093, 7.8396)	(0.0025, 0.3206)	(0.3288, 6.388)
	BAYS	(0.2921, 0.3078)	(0.4796, 0.5203)	(0.0951, 0.1048)	(1.9754, 2.0245)

**Table 15:** CI of estimates under MLE, MAP, and BAYS approaches with fixed values of $\varrho _1 = 1.8$, $\varrho _2 = 4.6$, $\rho = 2$, $\beta = 0.1$, $\zeta = 0.3$, $\phi = 0.5$, $p = 0.4$ for the Aircraft windshields data.

m	Method	${\hat{\zeta }}$	${\hat{\phi }}$	${\hat{\beta }}$	${\hat{\rho }}$
20	*MLE*	(0.1119, 2.9822)	(0.3799, 2.9957)	(0.0053, 0.1363)	(0.4054, 5.2887)
	MAP	(0.0892, 3.103)	(0.5585, 3.1174)	(0.0038, 0.1092)	(0.082, 5.1895)
	BAYS	(0.2853, 0.3146)	(0.4752, 0.5247)	(0.0924, 0.1075)	(1.9556, 2.0443)
22	*MLE*	(0.1016, 2.8635)	(0.4222, 2.905)	(0.0047, 0.1199)	(0.7824, 6.5019)
	MAP	(0.0772, 3.4976)	(0.463, 3.2578)	(0.003, 0.107)	(0.9809, 8.2249)
	BAYS	(0.2871, 0.3128)	(0.4803, 0.5196)	(0.0932, 0.1067)	(1.9604, 2.0395)
27	*MLE*	(0.1062, 2.9921)	(0.4439, 2.9777)	(0.005, 0.125)	(0.4945, 2.8993)
	MAP	(0.1507, 3.2796)	(0.1395, 3.9335)	(0.0064, 0.2114)	(0.6504, 5.8739)
	BAYS	(0.2894, 0.3105)	(0.4778, 0.5221)	(0.0941, 0.1058)	(1.9653, 2.0346)
30	*MLE*	(0.1097, 3.0361)	(0.4253, 3.0227)	(0.0051, 0.1312)	(0.5693, 3.0052)
	MAP	(0.1021, 3.073)	(0.3729, 3.2113)	(0.0042, 0.1372)	(0.6677, 3.9337)
	BAYS	(0.2867, 0.3132)	(0.4791, 0.5208)	(0.0938, 0.1061)	(1.9702, 2.0297)
40	*MLE*	(0.1188, 3.3614)	(0.3337, 3.3454)	(0.0051, 0.1614)	(0.3594, 1.858)
	MAP	(0.1332, 3.9441)	(0.1806, 4.4147)	(0.005, 0.2472)	(0.3112, 2.4004)
	BAYS	(0.2889, 0.3110)	(0.4765, 0.5234)	(0.0946, 0.1053)	(1.9751, 2.0248)

**Table 16:** Censoring schemes in the case of *SSPALT* under $PT\text {-}II CBRs$ for *n*=85 with fixed values of, $\varrho _1$ = 1.5, $\varrho _2$ = 4.5 and *p* = 0.4 for the Aircraft windshields data.

*i*	$m=$20				$m=$22				$m=$27				$m=$30				$m=$40			
	$X_{1i}$	$R_{1i}$	$X_{2i}$	$R_{2i}$	$X_{1i}$	$R_{1i}$	$X_{2i}$	$R_{2i}$	$X_{1i}$	$R_{1i}$	$X_{2i}$	$R_{2i}$	$X_{1i}$	$R_{1i}$	$X_{2i}$	$R_{2i}$	$X_{1i}$	$R_{1i}$	$X_{2i}$	$R_{2i}$
1	0.04	3	1.505	2	0.04	1	1.505	2	0.04	1	1.505	1	0.04	3	1.505	2	0.04	2	1.505	2
2	0.301	0	1.506	0	0.301	2	1.506	1	0.301	1	1.506	2	0.301	2	1.506	0	0.301	2	1.506	2
3	0.309	1	1.568	3	0.309	1	1.568	2	0.309	1	1.568	2	0.309	1	1.568	1	0.309	1	1.615	2
4	0.557	1	1.615	1	0.557	2	1.619	1	0.557	2	1.652	2	0.557	3	1.652	3	0.557	0	1.619	3
5	0.943	1	1.652	3	0.943	1	1.652	0	0.943	2	1.757	1	0.943	2	1.866	1	0.943	2	1.652	2
6	1.07	3	1.866	3	1.07	2	1.757	0	1.07	3	1.866	1	1.07	2	1.876	2	1.07	1	1.757	1
7	1.124	1	1.899	2	1.124	2	1.866	2	1.124	1	1.899	0	1.124	0	1.899	2	1.124	1	1.866	0
8	1.248	1	1.911	37	1.281	2	1.876	1	1.281	3	1.912	1	1.248	3	1.911	2	1.248	2	1.899	2
9	1.281	0			1.303	2	1.899	1	1.303	0	1.981	2	1.303	0	2.038	2	1.281	2	1.914	0
10	1.303	1			1.432	3	1.912	1	1.432	2	2.01	1	1.432	1	2.097	1	1.303	2	1.981	2
11	1.432	1			1.48	1	1.914	33			2.038	0	1.48	3	2.154	1	1.432	2	2.089	1
12	1.48	1									2.097	1			2.223	2			2.19	0
13											2.135	2			2.224	3			2.223	1
14											2.19	2			2.3	3			2.229	2
15											2.194	2			2.481	3			2.324	1
16											2.229	2			2.89	3			2.481	1
17											2.324	20			2.934	1			2.61	0
18															3.376	0			2.661	2
19															4.255	3			2.688	2
20																			3.117	0
21																			3.443	2

**Table 17:** Censoring schemes in the case of *SSPALT* under $PT\text {-}II CBRs$ for *n*=85 with fixed values of, $\varrho _1$ = 1.4, $\varrho _2$ = 4.2 and *p* = 0.4 for the Aircraft windshields data.

*i*	$m=$20				$m=$22				$m=$27				$m=$30				$m=$40			
	$X_{1i}$	$R_{1i}$	$X_{2i}$	$R_{2i}$	$X_{1i}$	$R_{1i}$	$X_{2i}$	$R_{2i}$	$X_{1i}$	$R_{1i}$	$X_{2i}$	$R_{2i}$	$X_{1i}$	$R_{1i}$	$X_{2i}$	$R_{2i}$	$X_{1i}$	$R_{1i}$	$X_{2i}$	$R_{2i}$
1	0.04	2	1.432	1	0.04	0	1.432	1	0.04	1	1.432	0	0.04	1	1.432	1	0.04	1	1.506	2
2	0.301	2	1.48	0	0.301	1	1.48	1	0.301	3	1.48	2	0.301	1	1.48	3	0.301	1	1.568	1
3	0.309	1	1.505	2	0.309	3	1.506	1	0.309	1	1.505	2	0.309	1	1.505	2	0.309	2	1.615	2
4	0.557	2	1.506	1	0.557	3	1.615	2	0.557	2	1.506	3	0.557	2	1.568	2	0.557	2	1.619	1
5	0.943	2	1.568	3	0.943	3	1.619	1	0.943	1	1.615	0	0.943	1	1.615	1	0.943	1	1.652	2
6	1.07	2	1.615	3	1.07	2	1.652	2	1.07	1	1.619	2	1.07	3	1.652	2	1.07	2	1.757	0
7	1.124	1	1.619	2	1.124	2	1.866	3	1.124	1	1.757	2	1.124	2	1.757	2	1.248	2	1.876	1
8	1.248	0	1.757	0	1.248	1	1.911	1	1.248	2	1.866	3	1.248	0	1.866	1	1.303	1	1.899	2
9	1.281	0	1.876	0	1.281	0	1.981	2	1.303	3	1.876	3	1.281	1	1.876	1			1.911	2
10	1.303	2	1.899	39		1.303	1	2.038	1			1.914	1	1.303	1	1.899	2		1.912	2
11							2.089	0			1.981	3			1.912	2			1.914	0
12							2.097	32			2.01	2			1.914	2			2.089	2
13											2.038	3			2.01	1			2.097	1
14											2.089	2			2.154	1			2.135	2
15											2.097	2			2.19	1			2.154	1
16											2.154	1			2.224	3			2.19	2
17											2.19	2			2.385	0			2.194	1
18											2.224	10			2.61	1			2.223	0
19															2.661	2			2.224	3
20															3.103	12			2.229	2
21																			2.61	1
22																			2.934	1
23																			2.964	0
24																			3.117	2

**Table 18:** Ceensoring schemes in the case of *SSPALT* under $PT\text {-}II CBRs$ for *n*=85 with fixed values of, $\varrho _1$ = 1.8, $\varrho _2$ = 4.6 and *p* = 0.4 for the Aircraft windshields data.

*i*	$m=$20				$m=$22				$m=$27				$m=$30				$m=$40			
	$X_{1i}$	$R_{1i}$	$X_{2i}$	$R_{2i}$	$X_{1i}$	$R_{1i}$	$X_{2i}$	$R_{2i}$	$X_{1i}$	$R_{1i}$	$X_{2i}$	$R_{2i}$	$X_{1i}$	$R_{1i}$	$X_{2i}$	$R_{2i}$	$X_{1i}$	$R_{1i}$	$X_{2i}$	$R_{2i}$
1	0.04	2	1.866	2	0.04	2	1.866	1	0.04	0	1.876	2	0.04	3	1.866	0	0.04	0	1.866	2
2	0.301	2	1.876	1	0.301	1	1.899	1	0.301	0	1.899	2	0.301	1	1.876	2	0.301	1	1.899	1
3	0.309	1	1.899	1	0.309	0	1.981	1	0.309	2	1.912	1	0.557	2	1.899	1	0.309	1	1.912	2
4	0.557	2	1.911	0	0.557	1	2.038	1	0.557	3	1.981	2	0.943	3	1.912	1	0.943	2	1.914	2
5	0.943	1	1.914	37	0.943	2	2.085	1	0.943	3	2.038	2	1.07	1	1.914	2	1.07	2	1.981	1
6	1.07	3			1.07	1	2.089	41	1.07	1	2.135	1	1.124	2	1.981	0	1.124	2	2.038	2
7	1.248	2			1.248	0			1.124	2	2.194	2	1.281	2	2.01	3	1.248	2	2.089	2
8	1.281	1			1.281	0			1.248	0	2.223	1	1.303	1	2.038	3	1.281	1	2.154	2
9	1.303	1			1.303	2			1.303	1	2.229	1	1.432	1	2.085	1	1.303	1	2.194	2
10	1.432	1			1.432	2			1.432	2	2.481	2	1.48	3	2.089	1	1.432	1	2.223	1
11	1.48	3			1.48	1			1.505	1	2.61	20	1.505	2	2.097	1	1.48	1	2.229	3
12	1.506	0			1.505	1			1.506	1			1.568	1	2.135	0	1.505	1	2.385	2
13	1.568	2			1.506	1			1.615	1			1.652	2	2.154	1	1.506	2	2.625	2
14	1.619	2			1.568	1			1.619	1			1.757	1	2.324	3	1.568	2	2.632	2
15	1.652	1			1.615	1			1.652	2					2.625	2			2.661	2
16							1.652	1			1.757	2			2.688	9			3.117	1
17																			3.443	2
18																			3.924	1

**Table 19:** Parameter estimates and goodness of fit measures of the considered models for the Aircraft windshields data.

Model	Estimators							$K-S (P value)$	*AIC*	*CAIC*	*BIC*	*HQIC*
Exponential	0.3902							0.3032 (0.0001)	331.9754	332.0236	334.418	332.9579
Weibull	3.5274	1.3765						0.1037 (0.3196)	280.7907	280.937	285.676	282.7557
Gamma	2.3931	0.0803						0.0535 (0.968)	266.5769	266.7232	271.4622	268.5419
Burr-XII	3.1791	0.3503						0.2758 (0.0001)	347.3611	347.5074	352.2464	349.3261
*MWD*	**1.1554**	**0.4932**	**0.0638**					**0.0873 (0.5361)**	**262.5203**	**262.8166**	**269.8482**	**265.4678**
GIWD	0.6593	1.9458	0.8401					0.3135 (0.0001)	399.9235	400.2198	407.2515	402.8711
Ex-weibuII	5.7806	0.2843	0.2538					0.0807 (0.6376)	263.5853	263.8816	270.9133	266.5329
KuE-weibuII	5.2934	0.0049	0.1542	0.3613				0.0632 (0.8866)	264.6624	265.1624	274.433	268.5924
IW-weibuII	1.9118	1.1204	88.0813	0.1976				0.0834 (0.5949)	269.3479	269.8479	279.1185	273.278
GIKw-Weibull	0.1138	1.8294	0.1241	0.8952	2.3462			0.0831 (0.6008)	266.2915	267.051	278.5048	271.2041
ExKu-weibuII	2.3743	0.7834	0.1595	0.127	1.8376			0.0872 (0.5382)	266.4985	267.258	278.7118	271.411
EGIKw-Weibull	0.21	2.0084	0.551	0.2946	1.36	2.0834		0.0822 (0.6132)	267.6675	268.7444	282.3234	273.5625

## Concluding remarks and future directions

This work addressed the issue of estimating *MWD* parameters under $PT\text {-}II CBRs$ samples for *SSPALT* in aviation engineering applications of aircraft windshield failure times. The results consistently revealed that, in all situations, the Bayesian methods yield stronger and more accurate parameter estimates. To solve the estimate problem with respect to balanced and unbalanced *GELF*, *LINEX*, and *SELF*, a Bayesian approach utilizing *MCMC* method in terms of *BGELF* was successful. The advantages of Bayesian credible intervals over normal CI in giving a larger scope of determining parameter uncertainty was also brought up in the research. By simulation experiments and applications of real data, the performance of the suggested estimation methods was verified. The application of the modified Weibull distribution provided more flexibility in the modeling of failure times and is thus a useful tool for reliability engineers and statisticians. In conclusion, for engineering applications, we advocate the Bayesian technique discussed in this paper to estimate MWD parameters based on SSPALT and PT-II CBR samples. The method ensures accuracy and dependability, a requirement for engineering reliability evaluation and maintenance planning.

Future work should aim at pushing the Bayesian method to more general reliability models, e.g., hierarchical Bayesian techniques or non-parametric Bayesian estimation. Another line of investigation can be on exploring other prior distributions and their influence on parameter inference. Future investigations could also incorporate MCMC techniques with advanced sampling schemes for better computational effectiveness. Lastly, implementing the suggested techniques in other disciplines, e.g., biomedical research and risk modeling in finance, would make the results more generalizable. Besides, future work could enhance this study on precise point estimation by incorporating bootstrap confidence intervals. This would address small-sample limitations of normal approximations, broadening practical utility.

## Data Availability

The datasets used and/or analysed during the current study available from the corresponding author on reasonable request

## Appendix

In this appendix, we present the expressions of the gradients needed for the implementation of various techniques discussed in this work. The *MLE* can be obtained by solving the following nonlinear likelihood equations

A.1 $$\begin{aligned} \nabla \pounds \left ( \Phi \right) =\begin{pmatrix} \frac{\partial \pounds \left ( \Phi \right) }{\partial \zeta } \\ \frac{\partial \pounds \left ( \Phi \right) }{\partial \phi } \\ \frac{\partial \pounds \left ( \Phi \right) }{\partial \beta } \\ \frac{\partial \pounds \left ( \Phi \right) }{\partial \rho } \end{pmatrix} = \begin{pmatrix} 0 \\ 0 \\ 0 \\ 0 \end{pmatrix}, \end{aligned}$$

where,

$$\begin{aligned} \dfrac{\partial \pounds \left ( \Phi \right) }{\partial \zeta }&= \sum _{i=1}^{n_{1}}\log x_{i}+\sum _{i=1}^{n_{1}}\dfrac{1}{ (\zeta +\phi x_{i})}- \beta \sum _{i=1}^{n_{1}} (r_{i}+1) \log x_{i} x_{i}^{\zeta }e^{\phi x_{i}} + \sum _{i=n_{1}+1}^{m} \log \wp _{{{{\rho x_{i}}}}} \\ &\quad +\sum _{i=n_{1}+1}^{m}\dfrac{1}{ \big (\zeta +\phi \wp _{{{{\rho x_{i}}}}}\big )}-\beta \sum _{i=n_{1}+1}^{m} (r_{i}+1)\log \wp _{{{{\rho x_{i}}}}}\wp _{{{{\rho x_{i}}}}}^{\zeta }e^{\phi \wp _{{{{\rho x_{i}}}}}}\\ \dfrac{\partial \pounds \left ( \Phi \right) }{\partial \phi }&= \sum _{i=1}^{n_{1}}\dfrac{x_{i}}{ (\zeta +\phi x_{i}) }- \beta \sum _{i=1}^{n_{1}} (r_{i}+1) x_{i}^{\zeta +1}e^{\phi x_{i}}+ \sum _{i=1}^{n_{1}}x_{i} +\sum _{i=n_{1}+1}^{m}\dfrac{\wp _{{{{\rho x_{i}}}}}}{\big (\zeta +\phi \wp _{{{{\rho x_{i}}}}}\big )}\\ &\quad - \beta \sum _{i=n_{1}+1}^{m} (r_{i}+1) \wp _{{{{\rho x_{i}}}}}^{\zeta +1}e^{\phi \wp _{{{{\rho x_{i}}}}}}+ \sum _{i=n_{1}+1}^{m} \wp _{{{{\rho x_{i}}}}}\\ \dfrac{\partial \pounds \left ( \Phi \right) }{\partial \beta }&= \dfrac{ m}{ \beta } - \sum _{i=1}^{n_{1}} (r_{i}+1) x_{i}^{\zeta }e^{\phi x_{i}} - \sum _{i=n_{1}+1}^{m} (r_{i}+1) \wp _{{{{\rho x_{i}}}}}^{\zeta }e^{\phi \wp _{{{{\rho x_{i}}}}}} \\ \dfrac{\partial \pounds \left ( \Phi \right) }{\partial \rho }&= \dfrac{ (m-n_{1})}{\rho } + (\zeta -1)\sum _{i=n_{1}+1}^{m} \dfrac{ (x_{i}-\varrho )}{\wp _{{{{\rho x_{i}}}}} } +\phi \sum _{i=n_{1}+1}^{m}\dfrac{ (x_{i}-\varrho )}{\big (\zeta +\phi \wp _{{{{\rho x_{i}}}}}\big ) }\\ &\quad +\phi \sum _{i=n_{1}+1}^{m} (x_{i}-\varrho )-\beta \sum _{i=n_{1}+1}^{m} (r_{i}+1) (x_{i}-\varrho ) (\zeta +\phi \wp _{{{{\rho x_{i}}}}} ) \wp _{{{{\rho x_{i}}}}}^{\zeta -1}e^{\phi \wp _{{{{\rho x_{i}}}}}} \end{aligned}$$

The components of $I_{ij}$ $i,j=1,2,3,4;$ can be obtained by using the following second derivatives of Eq.  (15)

$$\begin{aligned}\dfrac{\partial ^{2} \pounds \left ( \Phi \right) }{\partial \zeta ^{2}}&= -\sum _{i=1}^{n_{1}}\dfrac{1}{ (\zeta +\phi x_{i})^{2}}- \beta \sum _{i=1}^{n_{1}} (r_{i}+1) \big (\log x_{i}\big )^{2} x_{i}^{\zeta }e^{\phi x_{i}} \\ &\quad -\sum _{i=n_{1}+1}^{m}\dfrac{1}{ \big (\zeta +\phi \wp _{{{{\rho x_{i}}}}}\big )^{2}}-\beta \sum _{i=n_{1}+1}^{m} (r_{i}+1)\big (\log \wp _{{{{\rho x_{i}}}}}\big )^{2}\wp _{{{{\rho x_{i}}}}}^{\zeta }e^{\phi \wp _{{{{\rho x_{i}}}}}}\\ \dfrac{\partial ^{2} \pounds \left ( \Phi \right) }{\partial \zeta \partial \phi }= &\quad -\sum _{i=1}^{n_{1}}\dfrac{x_{i}}{ (\zeta +\phi x_{i})^{2} }- \beta \sum _{i=1}^{n_{1}} (r_{i}+1) \log x_{i} x_{i}^{\zeta +1}e^{\phi x_{i}} -\sum _{i=n_{1}+1}^{m}\dfrac{\wp _{{{{\rho x_{i}}}}}}{\big (\zeta +\phi \wp _{{{{\rho x_{i}}}}}\big )^{2}}\\ &\quad - \beta \sum _{i=n_{1}+1}^{m} (r_{i}+1) \log \wp _{{{{\rho x_{i}}}}} \wp _{{{{\rho x_{i}}}}}^{\zeta +1}e^{\phi \wp _{{{{\rho x_{i}}}}}} \\ \dfrac{\partial ^{2} \pounds \left ( \Phi \right) }{\partial \zeta \partial \beta }&= - \sum _{i=1}^{n_{1}} (r_{i}+1) \log x_{i} x_{i}^{\zeta }e^{\phi x_{i}} - \sum _{i=n_{1}+1}^{m} (r_{i}+1) \log \wp _{{{{\rho x_{i}}}}} \wp _{{{{\rho x_{i}}}}}^{\zeta }e^{\phi \wp _{{{{\rho x_{i}}}}}} \\ \dfrac{\partial ^{2} \pounds \left ( \Phi \right) }{\partial \zeta \partial \rho }&= \sum _{i=n_{1}+1}^{m} \dfrac{ (x_{i}-\varrho )}{\wp _{{{{\rho x_{i}}}}} } -\phi \sum _{i=n_{1}+1}^{m}\dfrac{ (x_{i}-\varrho )}{\big (\zeta +\phi \wp _{{{{\rho x_{i}}}}}\big )^{2} }\\ &\quad -\beta \sum _{i=n_{1}+1}^{m} (r_{i}+1) (x_{i}-\varrho ) \left ( 1+\log \wp _{{{{\rho x_{i}}}}}\big (\zeta +\phi \wp _{{{{\rho x_{i}}}}}\big ) \right) \wp _{{{{\rho x_{i}}}}}^{\zeta -1}e^{\phi \wp _{{{{\rho x_{i}}}}}} \\ \dfrac{\partial ^{2} \pounds \left ( \Phi \right) }{\partial \phi ^{2}}&= -\sum _{i=1}^{n_{1}}\dfrac{x_{i}^{2}}{ (\zeta +\phi x_{i})^{2} }- \beta \sum _{i=1}^{n_{1}} (r_{i}+1) x_{i}^{\zeta +2}e^{\phi x_{i}}-\sum _{i=n_{1}+1}^{m}\dfrac{\wp _{{{{\rho x_{i}}}}}^{2}}{\big (\zeta +\phi \wp _{{{{\rho x_{i}}}}}\big )^{2}}- \beta \sum _{i=n_{1}+1}^{m} (r_{i}+1) \wp _{{{{\rho x_{i}}}}}^{\zeta +2}e^{\phi \wp _{{{{\rho x_{i}}}}}} \\ \dfrac{\partial ^{2} \pounds \left ( \Phi \right) }{\partial \phi \partial \beta }&= - \sum _{i=1}^{n_{1}} (r_{i}+1) x_{i}^{\zeta +1}e^{\phi x_{i}} - \sum _{i=n_{1}+1}^{m} (r_{i}+1) \wp _{{{{\rho x_{i}}}}}^{\zeta +1}e^{\phi \wp _{{{{\rho x_{i}}}}}} \\ \dfrac{\partial ^{2} \pounds \left ( \Phi \right) }{\partial \phi \partial \rho }&= \sum _{i=n_{1}+1}^{m}\dfrac{ (x_{i}-\varrho )\big (\zeta +\wp _{{{{\rho x_{i}}}}} (\phi -1)\big )}{\big (\zeta +\phi \wp _{{{{\rho x_{i}}}}}\big )^{2} }+ \sum _{i=n_{1}+1}^{m} (x_{i}-\varrho )\\ &\quad -\beta \sum _{i=n_{1}+1}^{m} (r_{i}+1) (x_{i}-\varrho )\big (\zeta +\phi \wp _{{{{\rho x_{i}}}}}+1 \big ) \wp _{{{{\rho x_{i}}}}}^{\zeta }e^{\phi \wp _{{{{\rho x_{i}}}}}}\\ \dfrac{\partial ^{2} \pounds \left ( \Phi \right) }{\partial \beta ^{2}}&= -\dfrac{m}{\beta ^{2}} \\ \dfrac{\partial ^{2} \pounds \left ( \Phi \right) }{\partial \beta \partial \rho }&= -\sum _{i=n_{1}+1}^{m} (r_{i}+1) (x_{i}-\varrho ) (\zeta +\phi \wp _{{{{\rho x_{i}}}}} ) \wp _{{{{\rho x_{i}}}}}^{\zeta -1}e^{\phi \wp _{{{{\rho x_{i}}}}}} \\ \dfrac{\partial ^{2} \pounds \left ( \Phi \right) }{\partial \rho ^{2}}&= - \dfrac{ (m-n_{1})}{\rho ^{2} } - (\zeta -1)\sum _{i=n_{1}+1}^{m} \dfrac{ (x_{i}-\varrho )^{2}}{\wp _{{{{\rho x_{i}}}}}^{2} } -\phi ^{2}\sum _{i=n_{1}+1}^{m}\dfrac{ (x_{i}-\varrho )^{2}}{\big (\zeta +\phi \wp _{{{{\rho x_{i}}}}}\big )^{2} }\\ &\quad -\beta \sum _{i=n_{1}+1}^{m} (r_{i}+1) (x_{i}-\varrho )^{2}\left ( \zeta (\zeta -1)+\phi \wp _{{{{\rho x_{i}}}}}\big (2\zeta +\phi \wp _{{{{\rho x_{i}}}}} \big ) \right) \wp _{{{{\rho x_{i}}}}}^{\zeta -2}e^{\phi \wp _{{{{\rho x_{i}}}}}} \end{aligned}$$

The resulting Fisher Information Matrix for the Likelihood Eq.  (8) is:

A.2 $$\begin{aligned} I (\Phi ) = - \begin{bmatrix} \dfrac{\partial ^{2} \pounds \left ( \Phi \right) }{\partial \zeta ^{2}} & \quad \dfrac{\partial ^{2} \pounds \left ( \Phi \right) }{\partial \zeta \partial \phi } & \quad \dfrac{\partial ^{2} \pounds \left ( \Phi \right) }{\partial \zeta \partial \beta } & \quad \dfrac{\partial ^{2} \pounds \left ( \Phi \right) }{\partial \zeta \partial \rho } \\ \dfrac{\partial ^{2} \pounds \left ( \Phi \right) }{\partial \zeta \partial \phi } & \quad \dfrac{\partial ^{2} \pounds \left ( \Phi \right) }{\partial \phi ^{2}} & \quad \dfrac{\partial ^{2} \pounds \left ( \Phi \right) }{\partial \phi \partial \beta } & \quad \dfrac{\partial ^{2} \pounds \left ( \Phi \right) }{\partial \phi \partial \rho } \\ \dfrac{\partial ^{2} \pounds \left ( \Phi \right) }{\partial \zeta \partial \beta } & \quad \dfrac{\partial ^{2} \pounds \left ( \Phi \right) }{\partial \phi \partial \beta } & \quad \dfrac{\partial ^{2} \pounds \left ( \Phi \right) }{\partial \beta ^{2}} & \quad \dfrac{\partial ^{2} \pounds \left ( \Phi \right) }{\partial \beta \partial \rho } \\ \dfrac{\partial ^{2} \pounds \left ( \Phi \right) }{\partial \zeta \partial \rho } & \quad \dfrac{\partial ^{2} \pounds \left ( \Phi \right) }{\partial \phi \partial \rho } & \quad \dfrac{\partial ^{2} \pounds \left ( \Phi \right) }{\partial \beta \partial \rho } & \quad \dfrac{\partial ^{2} \pounds \left ( \Phi \right) }{\partial \rho ^{2}} \end{bmatrix} \end{aligned}$$

The corresponding Fisher Information Matrix for the joint prior Eq. 16 is:

A.3 $$\begin{aligned} I (\Phi ) = \begin{bmatrix} \frac{a_{1}-1}{\zeta ^2} & \quad 0 & \quad 0 & \quad 0 \\ 0 & \quad \frac{a_{2}-1}{\phi ^2} & \quad 0 & \quad 0 \\ 0 & \quad 0 & \quad \frac{a_{3}-1}{\beta ^2} & \quad 0 \\ 0 & \quad 0 & \quad 0 & \quad \frac{a_{4}-1}{\rho ^2} \end{bmatrix} \end{aligned}$$
